# Nanozyme‐Engineered Probiotic Microneedle Patch for Chronic Diabetic Wound Therapy

**DOI:** 10.1002/advs.202512127

**Published:** 2025-09-17

**Authors:** Xueyang Wang, Shuhua Liu, Shisuo Jing, Siyu Zhang, Yinli Jin, Wen Zhang, Qiongfang Ruan, Wei Li

**Affiliations:** ^1^ Department of Burns Tongren Hospital of Wuhan University (Wuhan Third Hospital) School of Pharmaceutical Sciences Wuhan University Wuhan 430071 P. R. China; ^2^ Taikang Center for Life and Medical Sciences Wuhan University Wuhan 430071 P. R. China; ^3^ Hubei Provincial Key Laboratory of Developmentally Originated Disease Wuhan 430071 P. R. China

**Keywords:** Diabetic wounds, Microneedles, Nanozymes, Probiotics

## Abstract

Chronic diabetic wounds are notoriously difficult to heal due to persistent bacterial infection, oxidative stress, and tissue hypoxia. Here, a multifunctional probiotic microneedle (MN) patch embedding platinum nanozyme‐engineered *Bacillus subtilis* (Pt‐M@*B. sub*) within a dissolvable, biocompatible polymer matrix is presented for synergistic diabetic wound therapy. This biohybrid system alleviates reactive oxygen species (ROS), combats bacterial infection, relieves hypoxia, and exerts anti‐inflammatory effects. The platinum nanozyme modification protects the probiotics of *B. subtilis* by scavenging ROS and generating oxygen in situ, thereby enhancing probiotic survival and function under harsh wound conditions. Upon transdermal application, the MN patch (termed Pt‐M@*B. sub* MN patch) enables efficient, minimally invasive delivery of the therapeutic nanozyme‐modified probiotics directly into the wound bed, where the functionalized living material significantly suppressed *Staphylococcus aureus* infection, reduced inflammation, promoted collagen deposition and angiogenesis, and accelerated wound closure. Biosafety assessments confirmed excellent biocompatibility and systemic safety of the probiotic MN patch. Moreover, the patch exhibited efficient skin penetration and drug delivery in human cadaver skin, indicating strong clinical translation potential. Overall, this work introduces a robust living‐materials‐based strategy that integrates microbial therapy with catalytic nanotechnology for effective management of chronic infected wounds.

## Graphical abstract

This work introduces a robust living‐materials‐based strategy that integrates microbial therapy with catalytic nanotechnology for effective management of chronic infected wounds via alleviating reactive oxygen species (ROS), combating bacterial infection, relieving hypoxia, and exerting anti‐inflammatory effects.

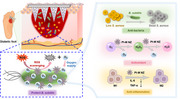

## 1 Introduction

As the global incidence of diabetes continues to rise, impaired wound healing, one of the most common complications of diabetes, has emerged as a leading cause of non‐traumatic amputation, imposing substantial psychological and economic burdens on patients.^[^ ^1^, ^2^, ^3^ ^]^ Normal wound healing progresses through four stages: hemostasis, inflammation, proliferation, and remodeling.^[^ ^4^, ^5^ ^]^ However, the complex microenvironmental factors in diabetic infected wounds lead to a prolonged wound healing phase.^[^ ^6^ ^]^ For instance, diabetic wounds often exhibit bacterial infections, resulting in the formation of bacterial biofilms that act as barriers to therapeutic drug penetration and significantly hinder the healing process.^[^ ^2^, ^7^, ^8^ ^]^ Moreover, the hyperglycemic microenvironment and bacterial infections in diabetic wounds trigger a dramatic increase in the production of reactive oxygen species (ROS), which can accumulate in the infected wounds, causing persistent oxidative stress and exacerbating inflammatory responses, prolonging the inflammatory phase.^[^ ^9^, ^10^ ^]^ Worse still, the hypoxic microenvironment in diabetic wounds further impairs angiogenesis, delaying the wound healing process.^[^ ^11^, ^12^ ^]^ Although current treatments, such as antibiotic therapy,^[^ ^13^ ^]^ hydrogel dressings,^[^ ^14^ ^]^ and hyperbaric oxygen therapy,^[^ ^15^ ^]^ have achieved some success in managing diabetic infected wounds, their widespread application remains limited by several critical issues. First, the overuse of antibiotics has led to the rise of antibiotic‐resistant bacteria, posing a global public health crisis.^[^ ^16^ ^]^ Second, the formation of bacterial biofilms in diabetic wounds restricts drug penetration into deeper tissues, significantly diminishing treatment efficacy.^[^ ^17^, ^18^ ^]^ Furthermore, existing therapies often target only a single aspect of wound healing, whereas diabetic infected wounds require a multifaceted approach. An ideal solution would involve a multifunctional system capable of alleviating oxidative stress and reducing inflammation at the wound site, while simultaneously delivering antimicrobial agents without inducing drug resistance.

Nanozymes (NZ) are a class of nanomaterials with enzyme‐like catalytic activity, capable of mimicking the functions of various enzymes, such as glutathione peroxidase (GPx),^[^ ^19^ ^]^ superoxide dismutase (SOD),^[^ ^20^ ^]^ peroxidase (POD),^[^ ^21^ ^]^ and catalase (CAT).^[^ ^22^ ^]^ Among these, SOD and CAT are particularly recognized for their critical roles in antioxidant defense.^[^ ^10^, ^23^ ^]^ Platinum nanozymes (Pt NZ), owing to their abundant active sites on the surface and unique electronic structure, exhibit CAT‐like and SOD‐like activities. These excellent biological activities ensure Pt NZ to effectively scavenge ROS and exert potent antioxidant effects.^[^ ^24^ ^]^ Additionally, Pt NZ demonstrates excellent biocompatibility and stability,^[^ ^25^ ^]^ making them highly attractive for biomedical applications. Pt NZ has been utilized in the treatment of various diseases, showcasing diverse biological activities such as anti‐tumor,^[^ ^26^ ^]^ anti‐inflammatory,^[^ ^27^ ^]^ and antioxidant.^[^ ^28^ ^]^ In the context of diabetic wound healing, the redox balance at the lesion site is closely related to wound healing and tissue reconstruction. Therefore, alleviating the oxidative stress state in this area is crucial for the treatment of diabetic wounds.^[^ ^10^, ^29^ ^]^ By exhibiting SOD and CAT activities, Pt NZ can remove excess superoxide anions (•O_2_^−^) and hydrogen peroxide (H_2_O_2_) in diabetic infected wounds, alleviating oxidative stress and inflammation. Moreover, Pt NZ generates oxygen during this process, addressing the hypoxic microenvironment and further promoting wound healing. These properties highlight the significant potential of Pt NZ in diabetic wound treatment.

The healing of skin wounds is closely related to microorganisms.^[^ ^30^ ^]^ Traditional antimicrobial drugs, while effective against pathogenic bacteria, often harm beneficial probiotics in the skin, disrupting the delicate balance of microflora at the wound site.^[^ ^31^ ^]^ In recent years, probiotics have attracted extensive attention in the field of wound healing as a novel antimicrobial agent.^[^ ^32^ ^]^ Probiotics exert their antimicrobial effects through multiple mechanisms, including the production of various antimicrobial substances and competition with harmful bacteria for nutrients and spatial resources.^[^ ^33^ ^]^ As natural biological agents, probiotics offer superior safety and tolerability compared to traditional antimicrobial drugs. More importantly, probiotics preserve the microflora balance at the wound site during the elimination of pathogenic bacteria, which is more conducive to the healing of diabetic wounds.^[^ ^34^ ^]^ Among probiotics, *Bacillus subtilis* (*B. subtilis*), an aerobic probiotic, has received widespread attention due to its ability to secrete antimicrobial peptides such as surfactin, which inhibit the growth of pathogens.^[^ ^35^ ^]^ While *B. subtilis* is known to produce extracellular proteases, these enzymes primarily function in nutrient acquisition and environmental adaptation.^[^ ^36^ ^]^ Recent studies have explored the application of *B. subtilis* in wound healing.^[^ ^37^, ^38^ ^]^ However, the high level of ROS and hypoxic microenvironment in diabetic infected wounds may adversely affect the activity of probiotics,^[^ ^39^, ^40^ ^]^ thereby limiting the therapeutic potential of *B. subtilis* in such conditions.

In recent years, microneedles (MNs), as a minimally invasive device that can penetrate the stratum corneum barrier of the skin to achieve transdermal drug delivery,^[^ ^41^ ^]^ have been widely applied in the fields of contraception,^[^ ^42^ ^]^ antitumor,^[^ ^43^ ^]^ and hair regeneration.^[^ ^44^ ^]^ The majority of bacterial infections induce biofilm formation, which are complex, attached microbial communities that form on surfaces or as aggregates embedded within an extracellular matrix.^[^ ^45^ ^]^ As a result, biofilms not only cover the wound surface, but are often hidden in the basal tissue, which creates antimicrobial barriers that restrict drug penetration, impair pathogen eradication, and compromise therapeutic outcomes. Unlike traditional drug delivery methods such as ointments and liniments, MNs can directly deliver drugs to the deep layers of the wound, thereby increasing the concentration of drugs at the lesion site.^[^ ^32^, ^46^, ^47^ ^]^ Furthermore, MNs interact minimally with dermal nerve endings, resulting in less pain, tissue damage, and skin inflammation for patients.^[^ ^48^ ^]^ These advantages make MNs‐based drug delivery a promising approach for the treatment of diabetic infected wounds, with significant clinical potential.

**Figure 1:**
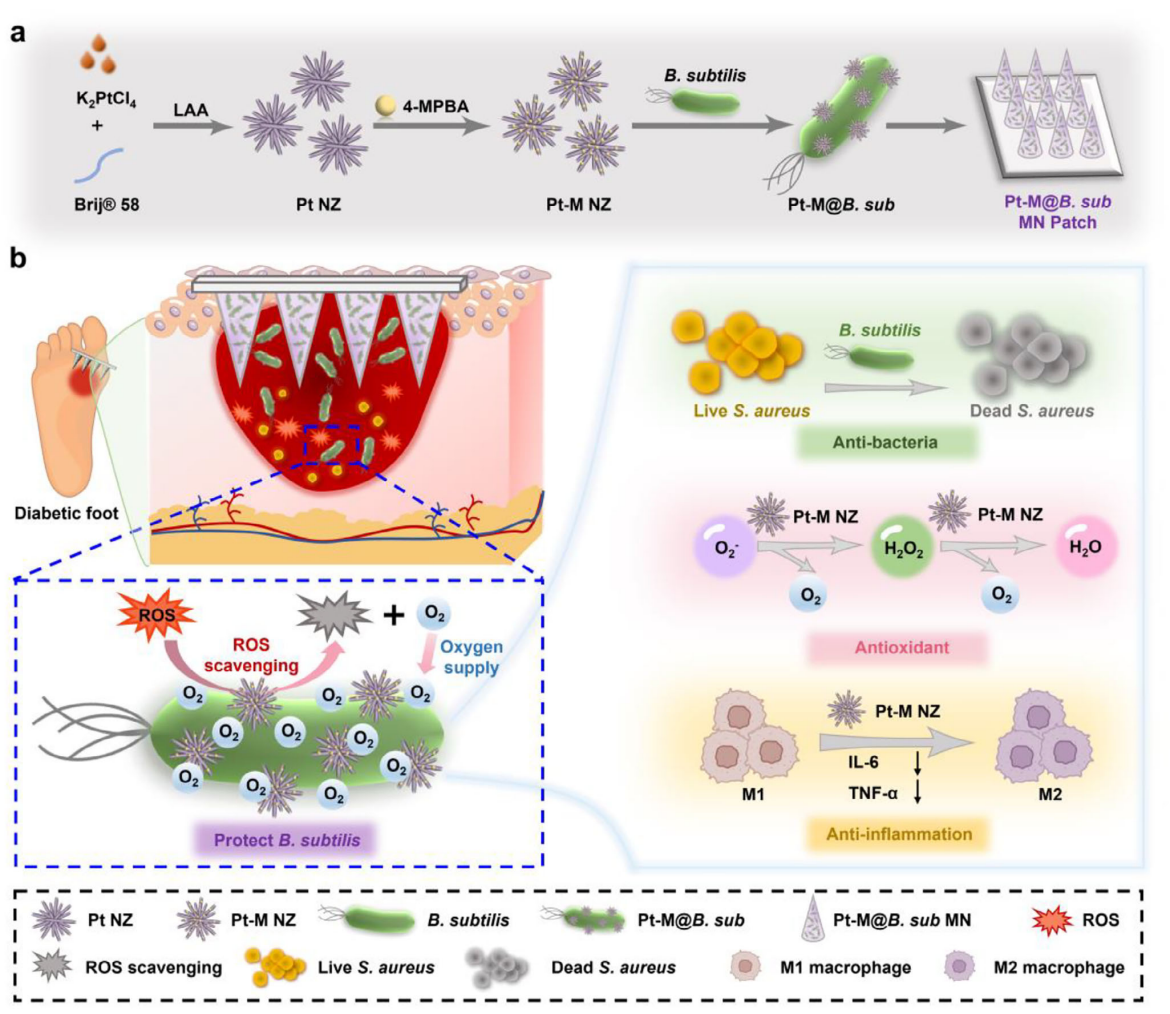
Schematic illustration of the fabrication and application of the nanozyme‐engineered probiotic MN patch (Pt‐M@*B. sub* MN patch) for the treatment of diabetic infected wounds. a) Fabrication process of the Pt‐M@*B. sub* MN patch. b) Multifunctional therapeutic mechanisms of the Pt‐M@*B. sub* MN patch in accelerating diabetic infected wounds healing.

To address the challenges and achieve the desired therapeutic goals, we designed a multifunctional MN patch loaded with nanozyme‐engineered probiotics for the treatment of diabetic infected wounds. Specifically, 4‐mercaptophenylboronic acid (4‐MPBA) is conjugate to Pt NZ via Pt‐S bonds to form Pt‐M NZ. The 4‐MPBA, owing to its boronic acid groups, reacts with the abundant vicinal diol structures on the bacterial surface to form boronic ester bonds, thereby modifying the Pt‐M NZ onto the surface of *B. subtilis* to form the nanozyme‐engineered probiotics (Pt‐M@*B. sub*), which are subsequently loaded into a water‐soluble MN patch (Pt‐M@*B. sub* MN patch) (**Figure** **1** **a)**. At the diabetic wound site, the MN patch rapidly dissolves, enabling efficient delivery of the Pt‐M@*B. sub*. The Pt‐M NZ component effectively mimics the activities of natural antioxidant enzymes (SOD and CAT) to scavenge •O_2_^−^ and H_2_O_2_, thereby exerting potent antioxidant and anti‐inflammatory effects. Simultaneously, the Pt‐M NZ protects *B. subtilis* by eliminating localized ROS and generating oxygen to *B. subtilis* in situ. This protective mechanism ensures that *B. subtilis* maintains its activity at the wound site, overcoming the challenge of probiotic survival under high ROS conditions and enhancing its antimicrobial efficacy (Figure 1b). Thus, the designed Pt‐M@*B. sub* MN patch achieves multifunctional therapeutic effects: *B. subtilis* provides robust antimicrobial activity, while the Pt‐M NZ significantly reduces ROS levels at the diabetic infected wound site, delivering antioxidant and anti‐inflammatory benefits. This innovative approach offers a promising strategy for the combined use of nanozymes and probiotics in the treatment of diabetic infected wounds.

## 2 Results

### 2.1 Synthesis and Characterization of Pt‐M NZ

**Figure 2:**
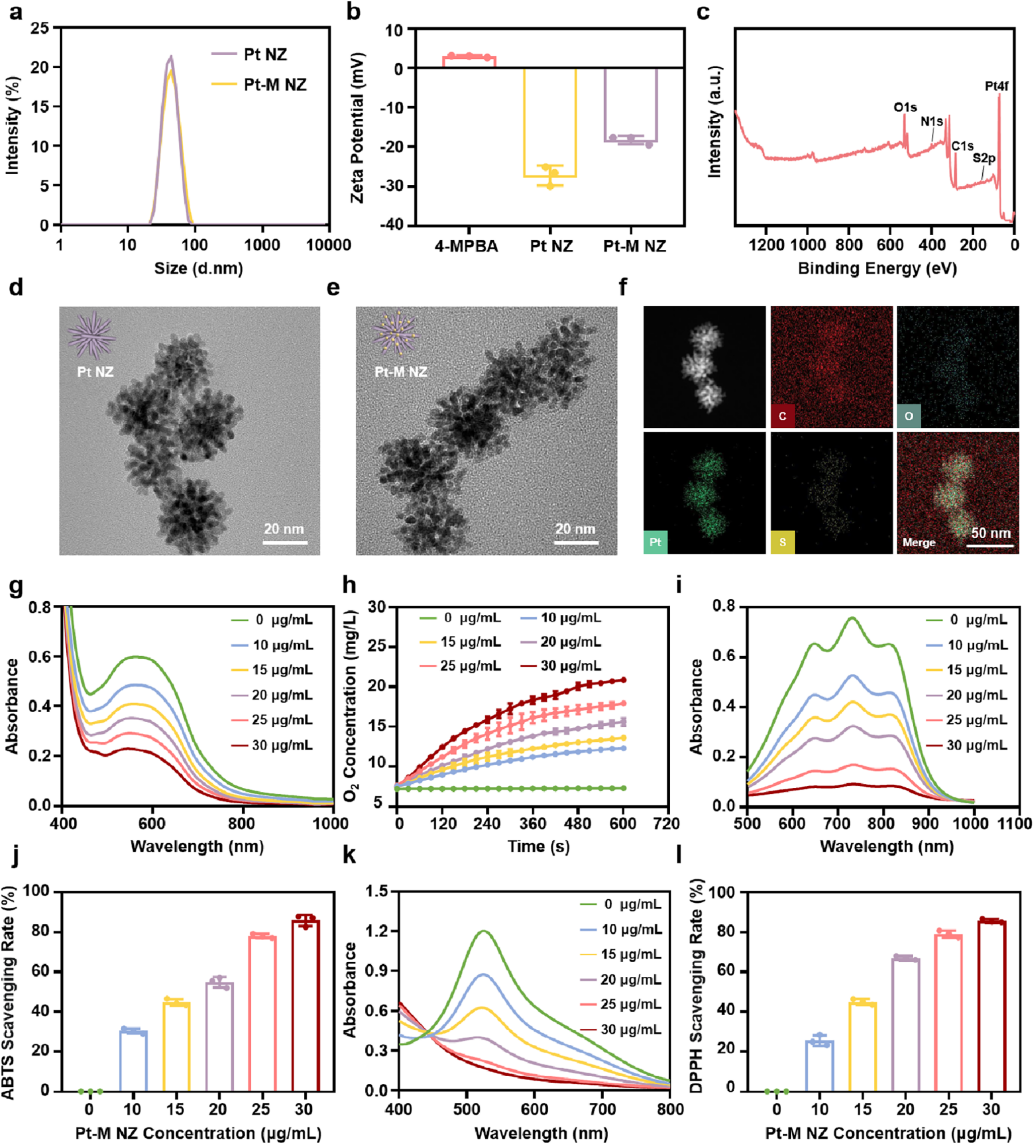
Characterization of Pt‐M NZ. a) Particle size distribution of Pt NZ and Pt‐M NZ measured by DLS. b) Zeta potential of 4‐MPBA, Pt NZ, and Pt‐M NZ. c) X‐ray photoelectron spectroscopy (XPS) spectra of Pt‐M NZ. Transmission electron microscopy (TEM) images of d) Pt NZ and e) Pt‐M NZ. f) TEM elemental mapping images of Pt‐M NZ. g) UV‐vis absorption spectra of nitroblue tetrazolium chloride (NBT) treated with different concentrations of Pt‐M NZ (0, 10, 15, 20, 25, 30 µg/mL) in glucose solution. h) Oxygen (O_2_) generation in H_2_O_2_ and glucose solution in the presence of different concentrations of Pt‐M NZ. i) UV‐vis absorption spectra of ABTS treated with different concentrations of Pt‐M NZ in glucose solution. j) ABTS scavenging rate at different concentrations of Pt‐M NZ in glucose solution. k) UV‐vis absorption spectra of DPPH treated with different concentrations of Pt‐M NZ in glucose solution. l) DPPH scavenging rate at different concentrations of Pt‐M NZ in glucose solution. Each point represents mean ± SD (n = 3).

The Pt NZ was synthesized through a one‐step reaction, followed by the attachment of 4‐MPBA to Pt NZ in a mixed ethanol‐water solution, forming Pt‐M NZ. Dynamic light scattering (DLS) analysis revealed that the particle size of Pt NZ was 40.63 ± 0.34 nm, while that of Pt‐M NZ was 41.32 ± 0.28 nm, indicating no significant size change following 4‐MPBA modification (**Figure** **2**a), which was consistent with transmission electron microscopy (TEM) observations (Figure 2d,e). The particle size of Pt‐M NZ remained stable over a 7‐day period, with the polydispersity index (PDI) consistently below 0.3, confirming the nanoparticles' excellent long‐term stability (Figure S1, Supporting Information). Zeta potential analysis showed a slight increase in the surface potential of Pt‐M NZ compared to Pt NZ after 4‐MPBA conjugation (Figure 2b), confirming the successful attachment of the positively charged 4‐MPBA molecules. The presence of sulfur (S), a characteristic element of 4‐MPBA, was confirmed by X‐ray photoelectron spectroscopy (XPS) (Figure 2c) and TEM elemental mapping (Figure 2f), further verifying the successful functionalization. Additionally, X‐ray diffraction (XRD) analysis demonstrated that the crystalline structure of Pt remained unchanged following 4‐MPBA conjugation (Figure S2, Supporting Information).

To evaluate the enzymatic activity and antioxidant capacity of Pt‐M NZ in diabetic wounds, subsequent enzyme activity and antioxidant assays were performed under conditions simulating hyperglycemic wound environments using 30 mm glucose solutions in vitro. The SOD‐like activity of Pt‐M NZ was evaluated using nitroblue tetrazolium chloride (NBT) as a probe. NBT can be reduced by •O_2_^−^ generated from riboflavin under light exposure to form blue formazan, characterized by a maximum absorption at 560 nm. As shown in Figure 2g, the absorption at 560 nm decreased with increasing concentrations of Pt‐M NZ, indicating that Pt‐M NZ inhibited the photoreduction of NBT in a concentration‐dependent manner and exhibited notable SOD‐like activity. CAT is a widely distributed antioxidant enzyme that catalyzes the decomposition of H_2_O_2_ into oxygen and water. The CAT‐like activity of Pt‐M NZ was assessed using a portable dissolved oxygen meter. As shown in Figure 2h, Pt‐M NZ facilitated a continuous, dose‐dependent production of oxygen upon incubation with H_2_O_2_, confirming its significant CAT‐like catalytic activity. The total antioxidant capacity of Pt‐M NZ was further evaluated using ABTS and DPPH free radical assays. As shown in Figure 2i,k, the absorbance peaks of both ABTS and DPPH progressively decreased with increasing Pt‐M NZ concentrations, indicating a positive correlation between the scavenging efficiency and Pt‐M NZ concentration. At a concentration of 30 µg mL^−1^, Pt‐M NZ scavenged 85.79% ± 2.33% of ABTS radicals (Figure 2j) and 85.48% ± 0.77% of DPPH radicals (Figure 2l), demonstrating its potent overall antioxidant capacity. Additionally, we evaluated changes in hydroxyl radical (•OH) and •O_2_^−^ levels mediated by Pt‐M NZ. In the •OH detection experiment, Fe^2+^ and H_2_O_2_ were used to generate •OH via the Fenton reaction as a positive control. Results revealed no significant color difference in solutions containing scavengers (salicylic acid or DPBF) between the Pt‐M NZ treatment group and the Control group, though vigorous bubbling occurred in the Pt‐M NZ solution; in contrast, the positive control exhibited pronounced color changes due to •OH generation (Figure S3a,b, Supporting Information). In the •O_2_^−^ scavenging experiment, Pt‐M NZ significantly eliminated •O_2_^−^ produced by light‐irradiated riboflavin (Figure S3c, Supporting Information). These results revealed that in H_2_O_2_‐rich environments, Pt‐M NZ predominantly displays CAT‐like activity by catalyzing H_2_O_2_ decomposition into oxygen without significant •OH generation. Additionally, Pt‐M NZ demonstrates effective •O_2_^−^ scavenging properties. These experiments demonstrated that Pt‐M NZ maintains robust SOD‐like and CAT‐like activities with potent antioxidant performance under simulated diabetic hyperglycemia.

### 2.2 Oxidative Stress Alleviation and Immunomodulatory Effects of Pt‐M NZ

**Figure 3:**
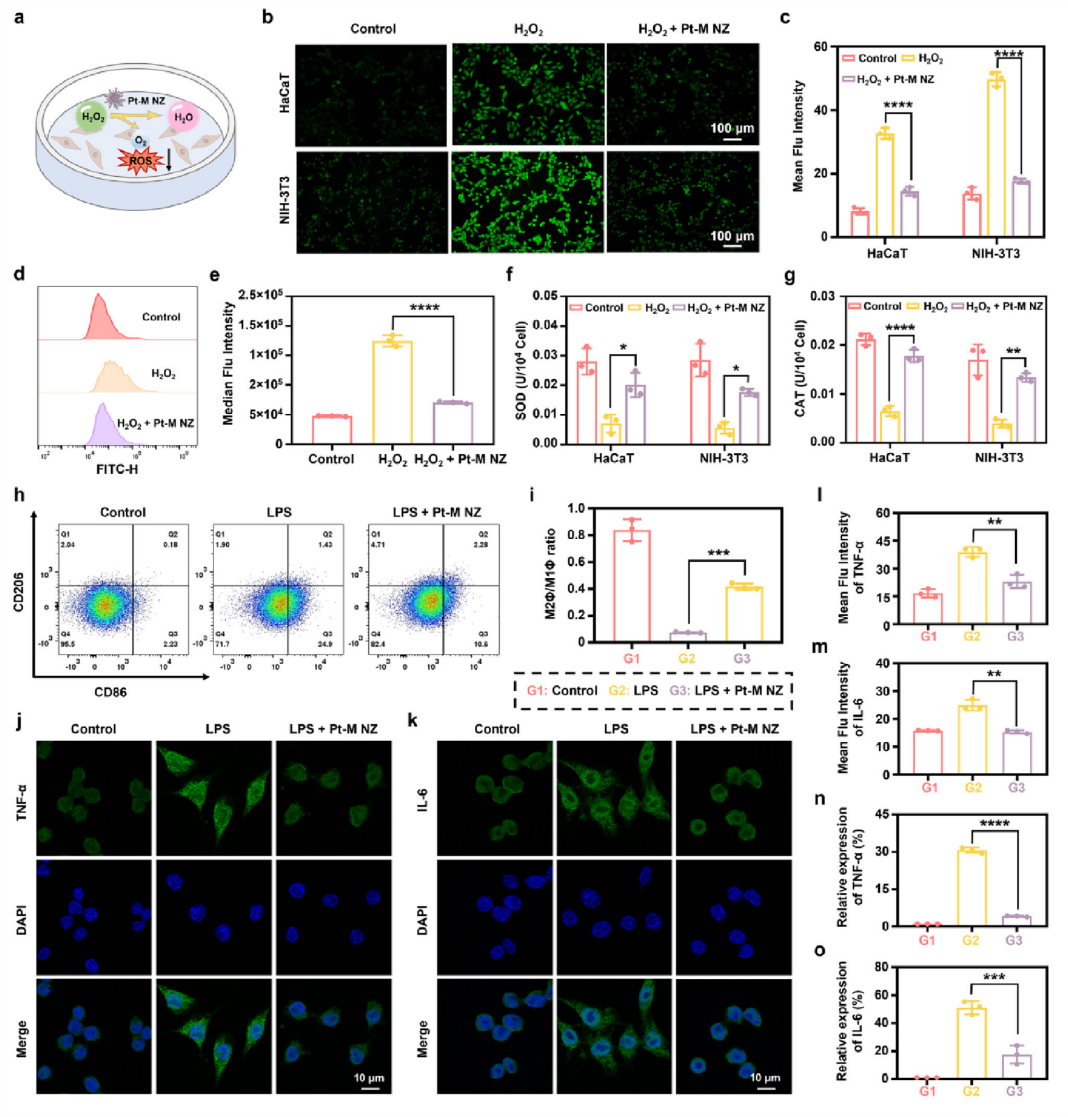
Antioxidant and anti‐inflammatory effects of Pt‐M NZ in vitro. a) Schematic illustration of Pt‐M NZ mitigating cellular oxidative stress through ROS scavenging. b) Representative ROS staining images and c) quantitative analysis of mean fluorescence intensity in HaCaT and NIH‐3T3 cells after incubation with DCFH‐DA probe. d) Flow cytometry results and e) quantitative analysis of median fluorescence intensity in HaCaT cells stained with DCFH‐DA under different treatments. f) SOD and g) CAT enzyme activities in HaCaT cells and NIH‐3T3 cells after different treatments. h) Flow cytometry analysis of macrophage polarization and i) quantitative M2Φ/M1Φ ratios under different treatments. j) Confocal laser scanning microscopy images and l) relative fluorescence intensity quantification of TNF‐α in RAW 264.7 cells. k) Confocal laser scanning microscopy images and m) relative fluorescence intensity quantification of IL‐6 in RAW 264.7 cells. Relative expression of n) TNF‐α and o) IL‐6 in RAW 264.7 cells determined by RT‐qPCR after different treatments. ^*^ *p* < 0.05, ^**^ *p* < 0.01, ^***^ *p* < 0.001, ^****^ *p* < 0.0001.

Diabetic infected wounds are characterized by elevated levels of ROS, which exacerbate cellular oxidative stress, leading to cellular damage and delayed wound healing. Consequently, ROS scavenging has emerged as a promising therapeutic strategy for managing diabetic wounds.^[^ ^49^ ^]^ The schematic image illustrates the regulatory mechanism of Pt‐M NZ in cells under high H_2_O_2_ conditions (**Figure** **3**a), showing its enzyme‐mimetic antioxidant activity in alleviating H_2_O_2_‐induced oxidative stress. To assess intracellular ROS levels, 2′,7′‐Dichlorodihydrofluorescein diacetate (DCFH‐DA), a fluorescent probe that converts to fluorescent 2,7‐dichlorofluorescein (DCF) upon ROS interaction, was utilized. As shown in Figure 3b,c, treatment with 400 µm H_2_O_2_ significantly increased fluorescence intensity in both Human keratinocytes (HaCaT) cells and mouse embryonic fibroblast (NIH‐3T3) cells, whereas pretreatment with Pt‐M NZ markedly attenuated this increase. Flow cytometry analysis further confirmed a significant reduction in fluorescence intensity in HaCaT cells (Figure 3d,e) and NIH‐3T3 cells (Figure S4, Supporting Information) following Pt‐M NZ pretreatment compared to the H_2_O_2_ group. Furthermore, Pt‐M NZ pretreatment significantly enhanced the activities of key antioxidant enzymes, including SOD (Figure 3f) and CAT (Figure 3g), in both HaCaT and NIH‐3T3 cells relative to H_2_O_2_ treated group. Collectively, these findings demonstrate the robust antioxidant capacity of Pt‐M NZ in protecting cells from exogenous oxidative stress.

In diabetic infected wounds, the impaired transition of macrophages from the pro‐inflammatory (M1) to the anti‐inflammatory (M2) leads to sustained elevation of pro‐inflammatory cytokines, thereby maintaining a chronic inflammatory environment that impedes wound healing. Pt‐M NZ exhibits anti‐inflammatory activity by restoring redox homeostasis through the elimination of ROS. Flow cytometry analysis revealed that lipopolysaccharide (LPS) stimulation significantly increased the proportion of M1 macrophages (CD86+ CD206‐), confirming successful polarization of M0 macrophages toward the M1 phenotype. Upon treatment with Pt‐M NZ, the proportion of M1 macrophages (CD86+ CD206‐) was significantly reduced, whereas the proportion of M2 macrophages (CD206+ CD86‐) increased. These results demonstrated that Pt‐M NZ effectively promoted repolarization from the M1 to the M2 phenotype, thereby exerting potent anti‐inflammatory effects (Figure 3h,i). Immunofluorescence staining of key inflammatory markers, including tumor necrosis factor‐alpha (TNF‐α) and interleukin‐6 (IL‐6), demonstrated increased fluorescence intensity following LPS stimulation. In contrast, Pt‐M NZ treatment significantly decreased the fluorescence intensity of TNF‐α (Figure 3j,l) and IL‐6 (Figure 3k,m). Furthermore, real‐time quantitative PCR (RT‐qPCR) confirmed that Pt‐M NZ significantly downregulated the mRNA expression of TNF‐α (Figure 3n) and IL‐6 (Figure 3o) in RAW 264.7 cells. Collectively, these findings demonstrate that Pt‐M NZ effectively mitigates LPS‐induced inflammation, underscoring its strong anti‐inflammatory potential.

### 2.3 Design and Characterization of Pt‐M@B. sub

**Figure 4:**
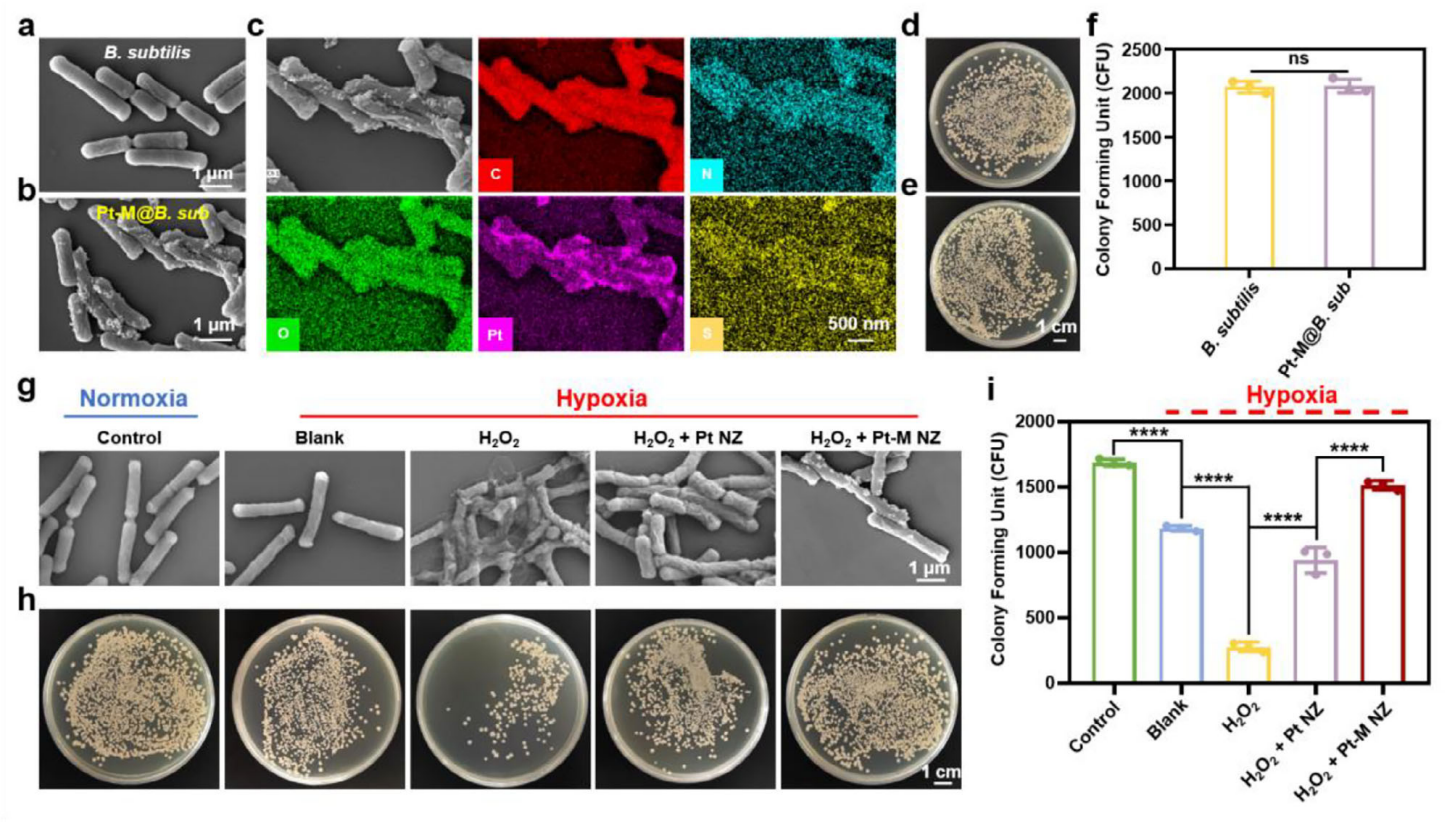
Characterization of Pt‐M@*B. sub*. SEM images of a) *B. subtilis* and b) Pt‐M@*B. sub*. c) EDS mapping images of the distribution of C, N, O, Pt and S elements in Pt‐M@*B. sub*. Representative photographs of d) *B. subtilis* and e) Pt‐M@*B. sub* on agar plates after 24 h incubation in PBS. f) Quantitative analysis of probiotics. g) SEM images of *B. subtilis* after different treatments. h) Photographs of the spread plate results and i) quantitative analysis of *B. subtilis* after different treatments. Each point represents mean ± SD (n = 3). ^****^ *p* < 0.0001. The ns indicates no significance.

We next immobilized the Pt‐M NZ onto the surface of *B. subtilis* through the interaction between the boric acid groups of 4‐MPBA and the vicinal diol structures on the bacterial surface, thereby forming a nanozyme‐engineered probiotic therapeutic agent (Pt‐M@*B. sub*). The coupling efficiency between Pt‐M NZ and *B. subtilis* was determined to be 89.76% ± 0.85% based on ICP‐MS analysis. Scanning electron microscopy (SEM) images confirmed the successful preparation of Pt‐M@*B. sub* (**Figure** **4**a,b), and SEM element mapping further verified the attachment of Pt‐M NZ on the surface of *B. subtilis* (Figure 4c). AFM‐IR results demonstrated a characteristic boronate ester bond peak at 1310 cm^−1^ at the Pt‐M@*B. sub* coupling interface, confirming covalent linkage between Pt‐M NZ's boronic acid groups and bacterial diol structures while providing robust spectroscopic evidence for stabilized spatial orientation through planar boronic acid/cis‐diol conformation matching (Figure S5, Supporting Information). The modification remained stable under oxidative stress, with no structural detachment observed after 24 h exposure to ROS (200 µm H_2_O_2_), as evidenced by SEM images (Figure S6, Supporting Information). This stability can be attributed to two synergistic mechanisms: (1) the intrinsic CAT‐ and SOD‐like activities of Pt‐M NZ, which effectively scavenge ROS and thereby protect boronate ester bonds from oxidative degradation, and (2) the 4‐MPBA‐modified surface, which enables the formation of multiple boronate ester bonds with bacterial diols. Even if partial bond cleavage occurs, the multivalent binding prevents detachment of Pt‐M NZ, ensuring robust and durable bacterial conjugation. To assess the impact of Pt‐M NZ modification on the viability of *B. subtilis*, both unmodified *B. subtilis* and Pt‐M@*B. sub* were incubated at 37 °C for 24 h and subsequently plated on Tryptose Soya Agar (TSA). Colony‐forming unit (CFU) counts indicated that the nanozyme modification did not compromise the viability of *B. subtilis* (Figure 4d–f). In addition to preserving viability, the nanozyme coating protected the probiotic from ROS‐induced damage, that is commonly elevated in diabetic infected wounds, and provided in situ oxygen generation, thereby significantly enhancing bacterial survival. The protective effects of Pt‐M@*B. sub* under hypoxic and ROS‐rich conditions were evaluated via SEM and the spread plate method. As shown in Figure 4g and *B. subtilis* exposed to H_2_O_2_ exhibited marked morphological damage. However, in the H_2_O_2_ + Pt NZ group, this damage was partially alleviated due to the ROS‐scavenging and oxygen‐generating capabilities of Pt NZ. Notably, in the H_2_O_2_ + Pt‐M NZ group, bacterial morphology was better preserved compared to the H_2_O_2_ + Pt NZ group, likely due to the localized ROS clearance and oxygen supply provided by the surface‐bound Pt‐M NZ. Spread plate analysis further confirmed that Pt‐M@*B. sub* significantly enhanced the survival of *B. subtilis* under high ROS and hypoxic conditions (Figure 4h,i).

### 2.4 Hypoxia Alleviation, Angiogenesis Promotion, and Cell Migration Enhancement by Pt‐M@B. sub

**Figure 5:**
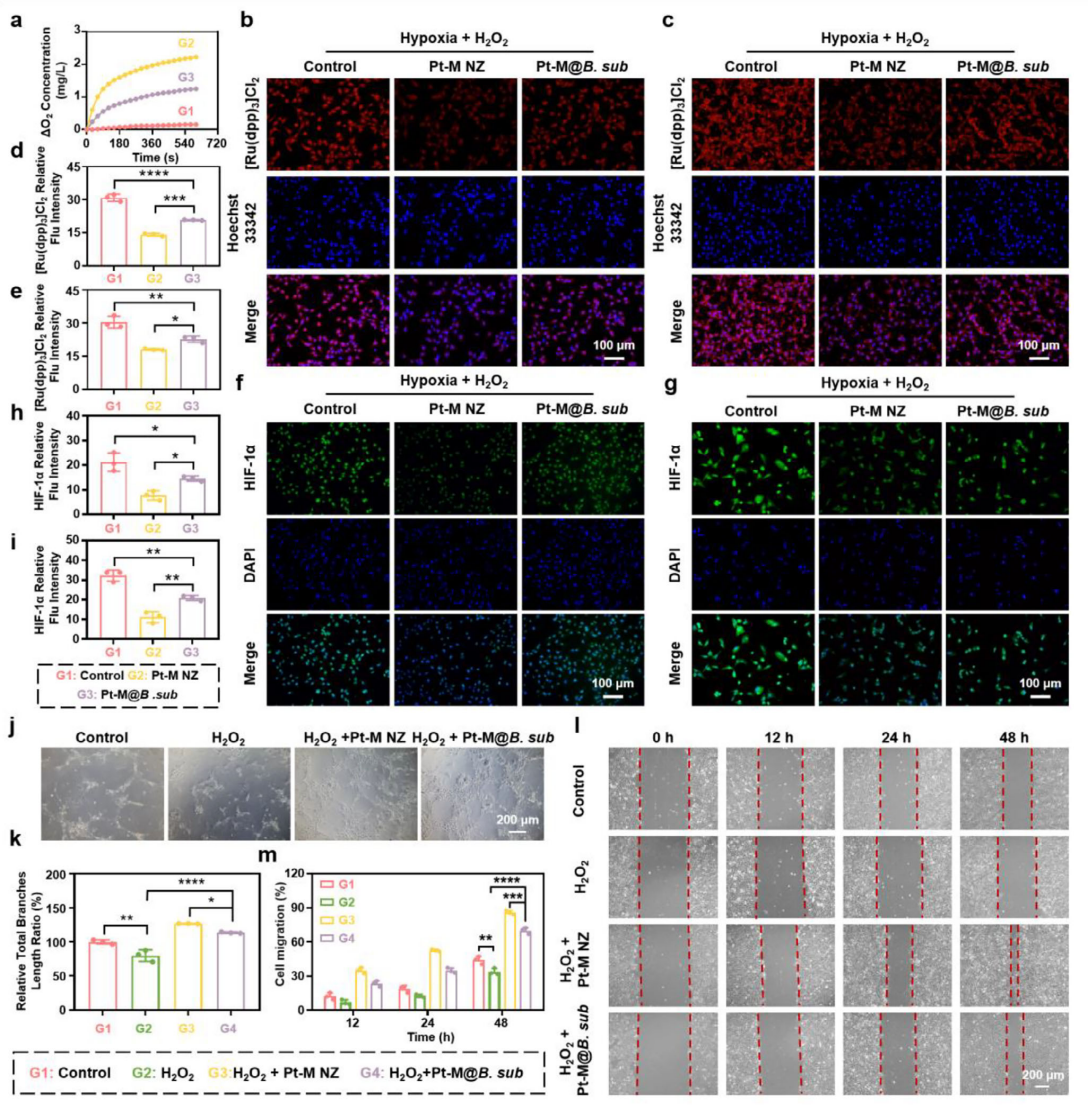
Hypoxia alleviation, angiogenesis promotion, and cell migration enhancement by Pt‐M@*B. sub*. a) Oxygen evolution capability of Pt‐M@*B. sub* in H_2_O_2_ solution under hypoxia conditions. b) Oxygen detection probe [Ru(dpp)_3_]Cl_2_ stained on HaCaT cells with different treatments and d) corresponding quantitative analysis. c) Oxygen detection probe [Ru(dpp)_3_]Cl_2_ stained on NIH‐3T3 cells with different treatments and e) corresponding quantitative analysis. f) Immunofluorescence staining images of HIF‐1α on HaCaT cells with different treatments and h) corresponding quantitative analysis. g) Immunofluorescence staining images of HIF‐1α on NIH‐3T3 cells with different treatments and i) corresponding quantitative analysis. j) Representative images of tube formation of HUVECs treated with different groups. k) Quantitative analysis of tube formation of HUVECs. l) Microscopy images and m) quantitative analysis of HaCaT cell migration under different treatments. Each point represents mean ± SD (n = 3). ^*^ *p* < 0.05, ^**^ *p* < 0.01, ^***^ *p* < 0.001, ^****^ *p* < 0.0001.

Utilizing the excellent CAT‐mimicking activity of Pt‐M NZ to catalytically H_2_O_2_ into oxygen, the efficacy of Pt‐M@*B. sub* in alleviating cellular hypoxia, promoting angiogenesis, and enhancing cell migration were investigated. Prior to these, oxygen production in solutions containing Pt‐M NZ or Pt‐M@*B. sub* was measured under in vitro hypoxic conditions with 400 µm H_2_O_2_ using a dissolved oxygen meter. The result showed that the oxygen production rate by Pt‐M NZ under hypoxic conditions with H_2_O_2_ supplementation exceeded the oxygen consumption by the immobilized *B. subtilis*, which led to a net increase in local oxygen availability, effectively alleviating hypoxia (**Figure** **5**a). Furthermore, in vitro studies using the oxygen‐sensitive fluorescent probe [Ru(dpp)_3_]Cl_2_ demonstrated significantly lower fluorescence intensity in the Pt‐M@*B. subtilis* group under hypoxic and ROS‐rich conditions, indicating higher oxygen levels (Figure 5b–e). This finding was further supported by reduced expression of hypoxia‐inducible factor‐1α (HIF‐1α), a cellular marker of hypoxia, in the same group (Figure 5f–i). Collectively, these data support that the Pt‐M@*B. Sub*system not only sustains the viability of the probiotic through self‐generated oxygen but also contributes to alleviating local tissue hypoxia.

Persistent oxidative stress impairs angiogenesis and suppresses cell migration, thereby delaying wound healing. The results in Figure 5j,k demonstrated that H_2_O_2_ treatment significantly inhibited the angiogenic capacity of HUVECs, while Pt‐M@*B. sub* treatment reversed this inhibition, confirming its potent pro‐angiogenic function. Cell migration of HaCaT cells under H_2_O_2_ stimulation was assessed via scratch assay. As shown in Figure 5l,m, H_2_O_2_ exposure markedly suppressed HaCaT cell migration, an effect counteracted by Pt‐M@*B. sub* treatment. These findings collectively indicate that Pt‐M@*B. sub* effectively mitigates ROS‐induced detrimental effects on both angiogenesis and cell migration.

### 2.5 Antibacterial Property of Pt‐M@B. sub In Vitro

**Figure 6:**
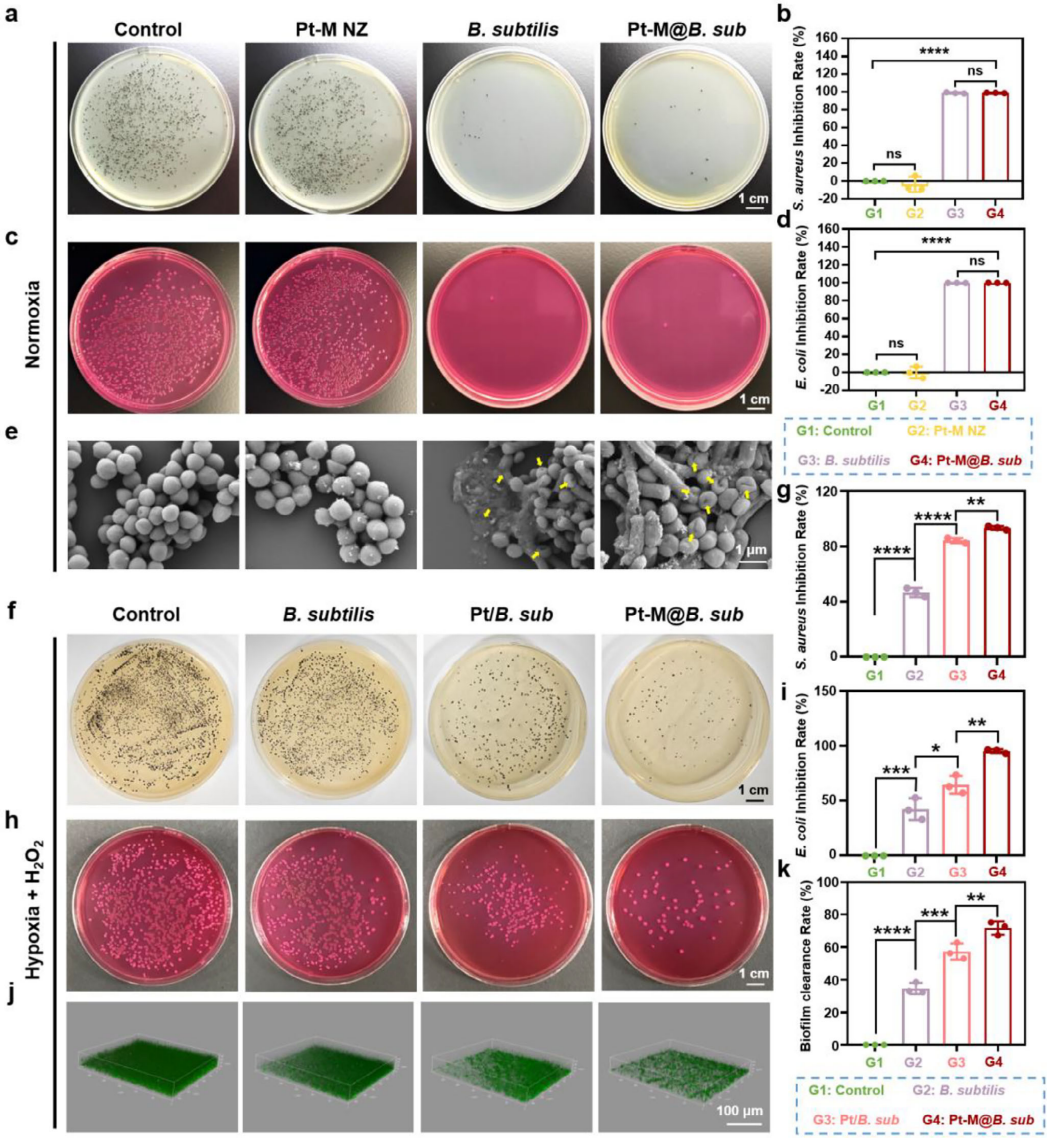
The antibacterial effect of Pt‐M@*B. sub* in vitro. a) Representative photographs and b) quantitative analysis of *S. aureus* colonies on agar plates after different treatments (Control, Pt‐M NZ, *B. subtilis*, and Pt‐M@*B. sub*). c) Representative photographs and d) quantitative analysis of *E. coli* colonies on agar plates after different treatments. e) Representative SEM images of *S. aureus* with different treatments. Yellow arrowheads indicate cracked or damaged *S. aureus*. f) Representative photographs and g) quantitative analysis of *S. aureus* colonies on agar plates after different treatments (Control, *B. subtilis*, Pt/*B. sub* and Pt‐M@*B. sub*) in the condition of hypoxia and H_2_O_2_. h) Representative photographs and i) quantitative analysis of *E. coli* colonies on agar plates after different treatments in the condition of hypoxia and H_2_O_2_. j) Confocal laser scanning microscopy and k) biofilm clearance rate of *S. aureus* after different treatments in the condition of hypoxia and H_2_O_2_. Green: live bacteria. Each point represents mean ± SD (n = 3). ^*^ *p* < 0.05, ^**^ *p* < 0.01, ^***^ *p* < 0.001, ^****^ *p* < 0.0001. The ns indicates no significance.

Diabetic wounds are frequently infected by *Staphylococcus aureus* (*S. aureus*) and *Escherichia coli* (*E. coli*). To assess the antibacterial efficacy of Pt‐M@*B. sub*, *S. aureus* and *E. coli* were selected as representative pathogens. Since *B. subtilis* can also grow on TSA plate, selective media were employed to distinguish *B. subtilis* from the target bacteria. For *S. aureus* isolation, 1% dipotassium trioxotellurate (DT) was added to TSA (TSA‐DT), which selectively inhibits most commensal bacteria while permitting *S. aureus* growth, resulting in distinct black‐pigmented colonies (Figure S7, Supporting Informationa). For *E. coli*, Desoxycholate (DC) Lactose Agar was utilized, leveraging its components (citric acid iron, sodium citrate, and sodium deoxycholate) to suppress Gram‐positive *B. subtilis* growth (Figure S7b, Supporting Information). *S. aureus* and *E. coli* were incubated with different treatments (untreated control, Pt‐M NZ, *B. subtilis*, and Pt‐M@*B. sub*) for 24 h before plating on their respective selective media. As shown in **Figure** **6**a,c, Pt‐M NZ alone did not exhibit significant antibacterial effects against either *S. aureus* or *E. coli*. In contrast, *B. subtilis* displayed strong antibacterial activity, with inhibition rates of 99.09% ± 0.54% for *S. aureus* and 99.79% ± 0.04% for *E. coli* (Figure 6b,d). Similarly, Pt‐M@*B. sub* achieved comparable inhibition rates of 99.15% ± 0.25% and 99.85% ± 0.04% for *S. aureus* and *E. coli*, respectively. Furthermore, morphological analysis via SEM revealed that *S. aureus* treated with Pt‐M NZ retained its typical spherical shape and intact cell membrane. In contrast, cells treated with *B. subtilis* or Pt‐M@*B. sub* exhibited pronounced membrane shrinkage and rupture (Figure 6e). These results indicate that *B. subtilis* exerts potent antibacterial activity against both *S. aureus* and *E. coli*, and that surface modification with Pt‐M NZ does not compromise its antimicrobial function in vitro.

The elevated levels of ROS and hypoxic conditions characteristic of diabetic infected wounds may compromise *B. subtilis* viability, thereby reducing its antimicrobial efficacy. To investigate this, an in vitro model was developed to mimic pathological concentrations of H_2_O_2_ and hypoxia, allowing assessment of the antibacterial performance of Pt‐M@*B. sub*. The results demonstrated that both Pt/*B. sub* (Pt NZ mixing with *B. subtilis*) and Pt‐M@*B. sub* exhibited significantly enhanced inhibitory effects against *S. aureus* (Figure 6f) and *E. coli* (Figure 6h) compared to unmodified *B. subtilis*. This enhancement was attributed to the platinum‐based nanozymes’ capacity to scavenge ROS and generate oxygen, thereby supporting bacterial viability and antimicrobial activity under stress conditions. Importantly, Pt‐M@*B. sub* showed superior antibacterial efficacy relative to Pt/*B. sub*, which was likely due to the localized ROS neutralization and targeted oxygen delivery enabled by surface‐modified Pt‐M NZ at the microbial interface (Figure 6g,i). Given the pivotal role of bacterial biofilms in the chronicity of diabetic wounds, we further evaluated the biofilm‐disrupting capability of Pt‐M@*B. sub*. As shown in Figure 6j, treatment with Pt‐M@*B. sub* led to a 71.77% ± 3.36% reduction in *S. aureus* biofilms (Figure 6k), confirming its potent antibacterial and antibiofilm activity under simulated wound conditions.

### 2.6 Fabrication and Characterization of Pt‐M@B. sub MN Patch

**Figure 7:**
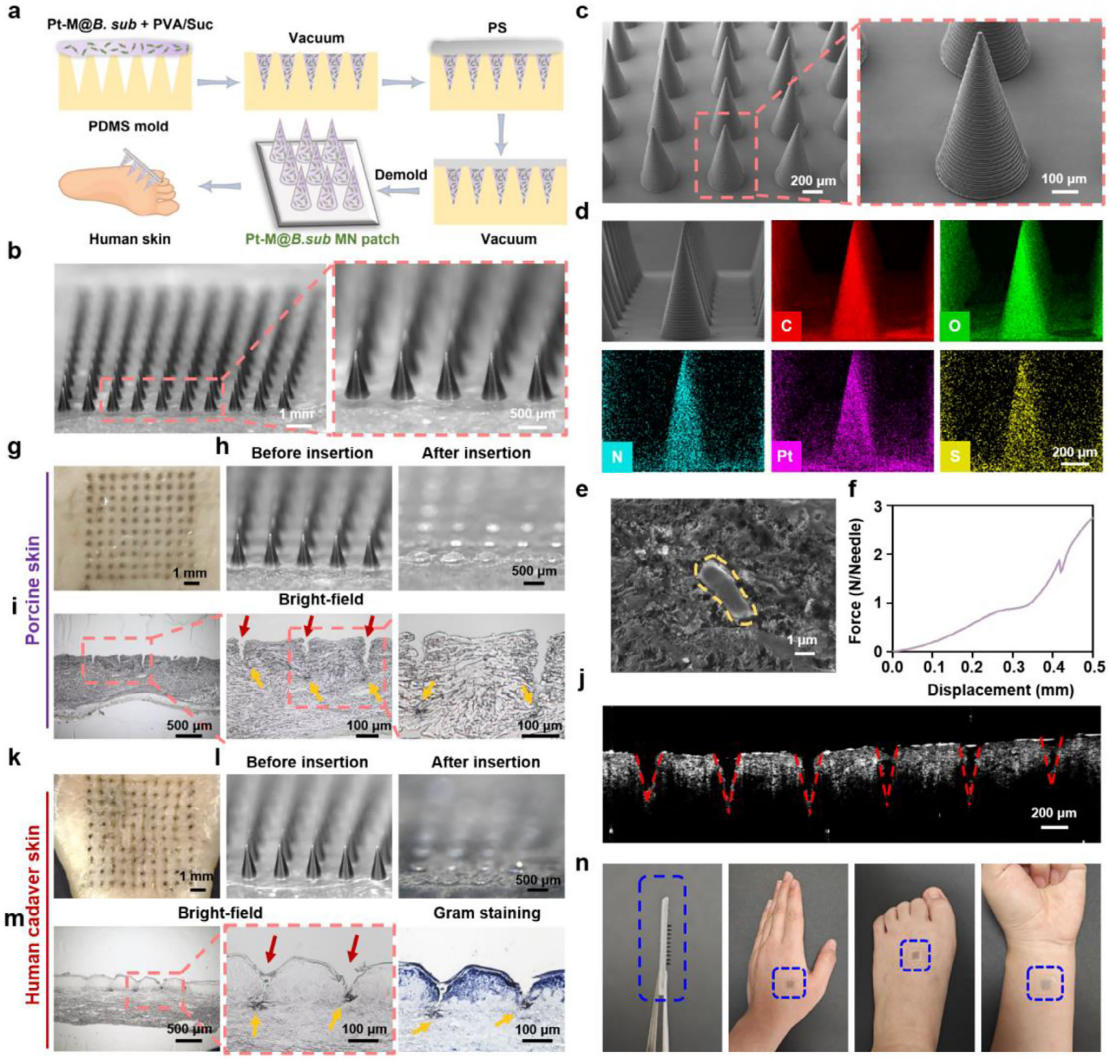
Characterization of Pt‐M@*B. sub* MN patch. a) Schematic illustration of the fabrication process of Pt‐M@*B. sub* MN patch. b) Optical images and c) SEM images of Pt‐M@*B. sub* MN patch. d) EDS elemental mapping images of the distribution of C, O, N, Pt and S elements in Pt‐M@*B. sub* MN. e) SEM image of a cross‐section of Pt‐M@*B. sub* MN showing the encapsulated probiotic. f) Force‐displacement curve of Pt‐M@*B. sub* MN. g) Bright‐field microscopy image of porcine skin after Pt‐M@*B. sub* MN patch insertion. h) Representative bright‐field images of one MN patch before and after insertion into porcine skin *ex vivo*. i) Histological images of porcine skin after MN patch application (red arrows indicate MN patch penetration sites; yellow arrows designate Pt‐M@*B. sub*). j) Optical coherence tomography (OCT) scanning image of the porcine skin punctured by MN patch. k) Bright‐field microscopy image of human cadaver skin after Pt‐M@*B. sub* MN patch insertion. l) Representative bright‐field images of one MN patch before and after insertion into human cadaver skin. m) Histological images and Gram‐staining image of human cadaver skin after MN patch application (red arrows indicate MN patch penetration sites; yellow arrows designate Pt‐M@*B. sub*). n) Demonstration photographs of the wearable MN patch attaching on the human hand, foot or arm without skin puncture. Blue dashed squares indicate the position of the MN patch.

Polyvinyl alcohol (PVA) and polystyrene (PS), both recognized for their excellent biocompatibility, were employed as matrix materials alongside sucrose, which enhances the mechanical strength of MNs, to fabricate the Pt‐M@*B. sub* MN patch via vacuum casting in a polydimethylsiloxane (PDMS) mold (**Figure** **7**a). Each MN patch contained a load of 37.34 µg Pt‐M NZ and 1.04 × 10^8^ CFU of *B. subtilis* as the Pt‐M@*B. sub* complex. As shown in Figure 7b, the resulting patch consisted of a 10 × 10 array of conical MNs, each with a height of 850 µm and a base diameter of 400 µm, as observed in SEM images (Figure 7c). Energy‐dispersive X‐ray spectroscopy (EDS) elemental mapping confirmed the uniform distribution of C, O, N, Pt, and S throughout the MN matrix, indicating successful incorporation and dispersion of Pt‐M@*B. sub* (Figure 7d). SEM imaging further confirmed that *B. subtilis* was effectively encapsulated within the MNs while maintaining its structural integrity (Figure 7e). Plate spreading results demonstrated that *B. subtilis* maintained approximately 80% viability following Pt‐M@*B. sub* MN patch fabrication (Figure S8, Supporting Information). The fabricated MNs retained effective viability of *B. subtilis* for over 7 days when being stored at 4 °C (Figure S9, Supporting Information). Mechanical testing revealed that the Pt‐M@*B. sub* MNs exhibited a failure force of approximately 1.88 N (Figure 7f), significantly exceeding the minimum force of 0.058 N required for effective skin penetration,^[^ ^50^ ^]^ thereby confirming their suitability for transdermal delivery. Upon application to *ex vivo* porcine skin for 5 min, the MNs rapidly dissolved (Figure 7h), enabling efficient delivery of Pt‐M@*B. sub* into the tissue (Figure 7g). Microscope images of porcine skin sections post‐application confirmed successful transdermal delivery: red arrows indicate MN patch penetration sites; yellow arrows designate Pt‐M@*B. sub* (Figure 7i). Optical coherence tomography (OCT) further validated effective MN penetration (Figure 7j). To assess clinical applicability, the MN patch was tested on human cadaver skin. As shown in Figure 7l, the MNs fully dissolved within 5 min, facilitating the delivery of Pt‐M@*B. sub* into human skin tissue (Figure 7k). Histological analysis of the treated tissue confirmed successful delivery, with MN insertion sites clearly visible (Figure 7m). Furthermore, the drug release rate of the Pt‐M@*B. sub* MN patch was investigated in diabetic infected wounds of rats. Results demonstrated complete dissolution of the MN tips within 3 min at the wound site, ensuring efficient release of the payload of Pt‐M@*B. sub* (Figure S10, Supporting Information). Importantly, the compact design of the MN patch allows convenient application to the human hand, foot, or arm, supporting its potential for practical therapeutic use (Figure 7n). Moreover, the Pt‐M@*B. sub* MN patch exhibited no significant cytotoxicity toward HaCaT and NIH‐3T3 cells, as demonstrated by cell viability assays (Figures S11,S12, Supporting Information), indicating excellent in vitro biocompatibility. This finding was further supported by a hemolysis assay, which confirmed the hemocompatibility of the patch (Figure S13, Supporting Information).

### 2.7 Therapeutic Efficacy of Pt‐M@B. sub MN Patch In Vivo

**Figure 8:**
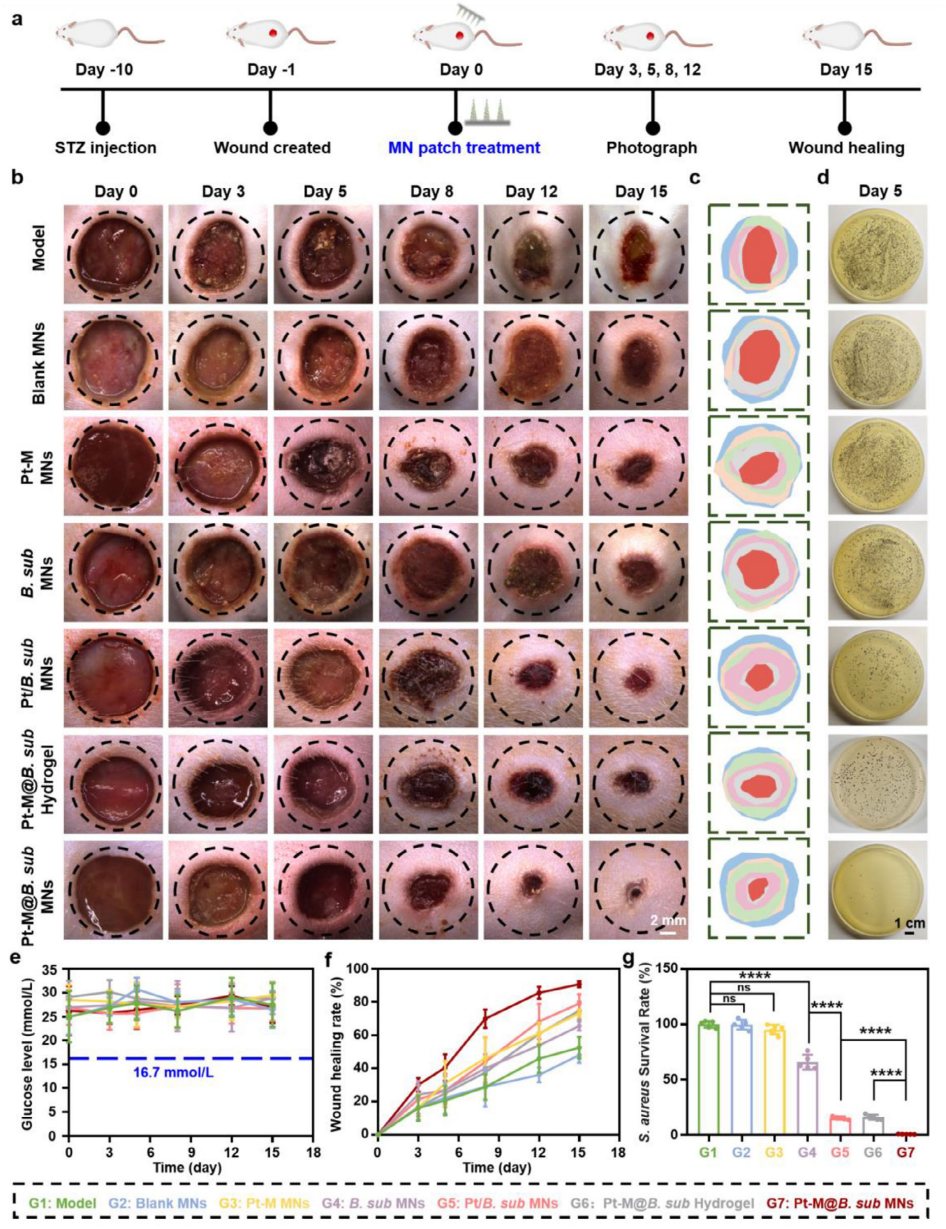
Antimicrobial analysis and wound healing assessment of Pt‐M@*B. sub* MN patch in vivo. a) Schematic illustration of diabetic infected wounds animal model construction and drug treatment protocol. b) Representative images of wound healing at different time points across treatment groups. c) Simulated schematic images of diabetic infected wounds changes over time with different treatments. d) Representative images of *S. aureus* colony formation on agar plates from wound tissues on day 5. e) Blood glucose levels in rats during the 15‐day healing period. f) Quantitative statistics of wound healing rates in different treatment groups. g) Quantitative statistics of *S. aureus* colony counts on day 5 in wound tissue of different treatments. Each point represents mean ± SD (n = 5). ^****^ *p* < 0.0001. The ns indicates no significance.

To evaluate the wound healing efficacy of Pt‐M@*B. sub* MN patches in vivo, a diabetic infected wound model was established in rats via intraperitoneal injection of streptozotocin (STZ). Full‐thickness circular wounds (1.0 cm in diameter) were created on the dorsal skin and subsequently infected with *S. aureus* (**Figure** **8**a). Rats were randomly assigned to seven treatment groups (Model, Blank MNs, Pt‐M MNs, *B. sub* MNs, Pt/*B. sub* MNs, Pt‐M@*B. sub* Hydrogel, and Pt‐M@*B. sub* MNs), with five rats per group (n = 5). Blood glucose levels were monitored throughout the study and consistently exceeded 16.7 mmol L^−1^, confirming the successful induction of a diabetic model (Figure 8e). Wound healing progression was monitored via photographic documentation on days 0, 3, 5, 8, 12, and 15 (Figure 8b), and simulated images were generated to visualize changes in wound size over time (Figure 8c). Quantitative analysis revealed that the Pt‐M@*B. sub* MNs group exhibited the most effective wound healing among all treatment groups (Figure 8f). To assess the antibacterial performance of the MN patches, *S. aureus* isolated from wound tissue on day 5 was cultured on TSA‐DT agar plates. As shown in Figure 8d,g, the *B. sub* MNs group exhibited limited antibacterial activity due to the hypoxic and oxidative conditions characteristic of diabetic wounds. In contrast, the Pt‐M@*B. sub* MNs group achieved near‐complete eradication of *S. aureus*. Notably, the Pt‐M@*B. sub* MNs group outperformed the Pt/*B. sub* MNs group, attributed to the surface modification of *B. subtilis* with Pt‐M NZ. After application, *B. subtilis* in the Pt‐M@*B. sub* MN treatment group survived longer in diabetic infected wounds (over 12 days) and exhibited enhanced clearance of *S. aureus* compared to the Pt/*B. sub* MN group (Figure S14, Supporting Information). This modification enabled ROS scavenging, protecting *B. subtilis* from oxidative stress, and simultaneously provided in situ oxygen generation, enhancing bacterial survival and therapeutic efficacy. This finding also indicated that our nanozyme‐engineered probiotic system does not lead to sustained colonization or uncontrolled bacterial proliferation, thereby supporting its favorable biosafety profile. Furthermore, the Pt‐M@*B. sub* Hydrogel‐treated group demonstrated inferior wound healing efficacy compared to the Pt‐M@*B. sub* MNs group, likely attributable to its inability to penetrate the bacterial biofilm barrier in diabetic infected wounds, resulting in reduced drug delivery efficiency. Collectively, these findings highlight the significant therapeutic potential of the Pt‐M@*B. sub* MN patch for the treatment of diabetic infected wounds.

### 2.8 Evaluation of Tissue Regeneration, Inflammatory Response, and Angiogenesis Post‐Treatment In Vivo

**Figure 9:**
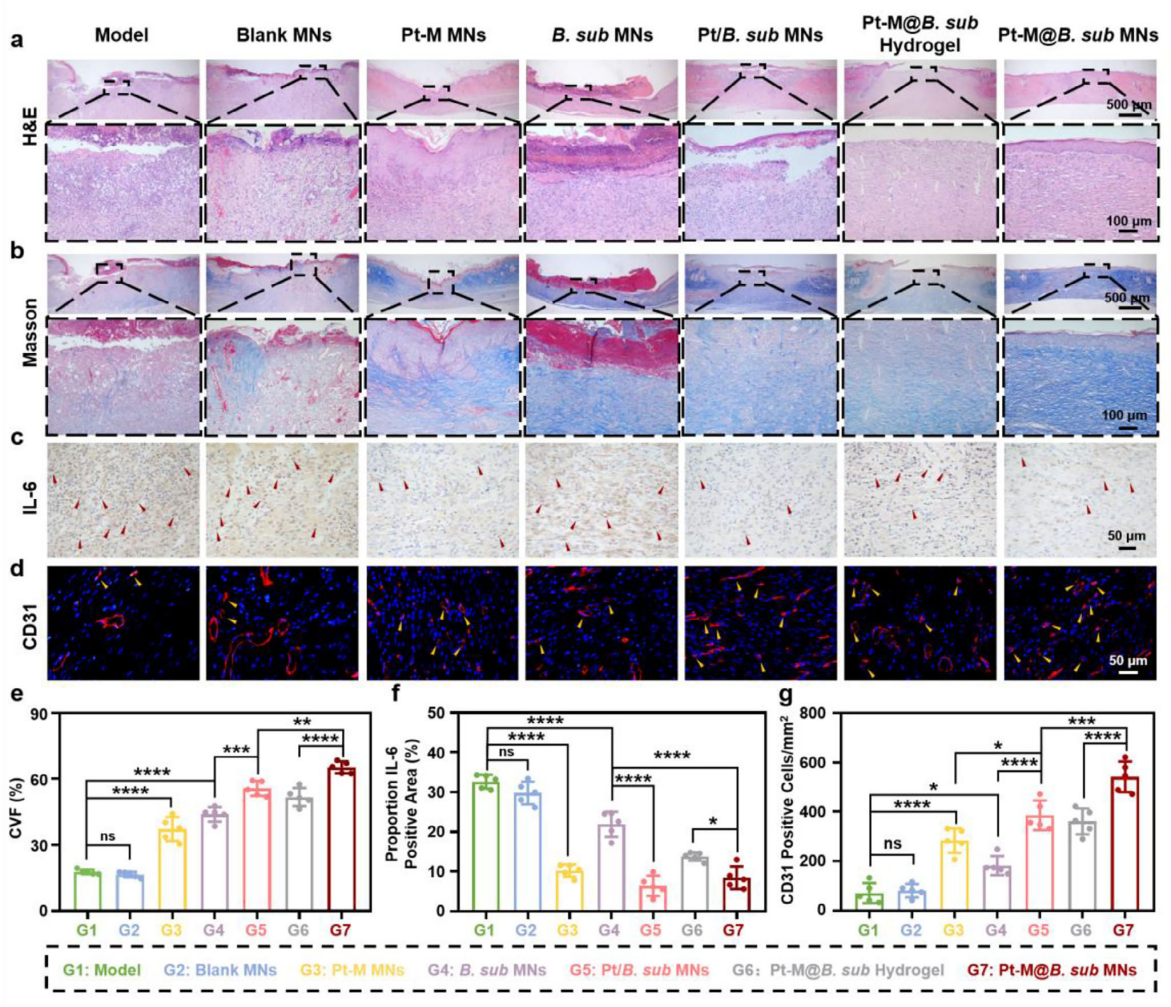
Histopathological analysis of diabetic infected wounds on day 15 post‐treatment. Representative bright‐field microscopy images of a) H&E staining, b) Masson staining, and c) immunohistochemistry staining of IL‐6 after different treatments (Red arrows indicate IL‐6‐positive cells). d) Representative immunofluorescence microscopy images of CD31 after different treatments (Yellow arrows indicate sites of neovascularization). e) Quantitative analysis of collagen volume fraction (CVF) for different treatments. f) Quantitative analysis of positive areas for IL‐6 immunohistochemical staining. g) Quantitative analysis of the relative coverage area of CD31 for different treatments. Each point represents mean ± SD (n = 5). ^*^ *p* < 0.05, ^**^ *p* < 0.01, ^***^ *p* < 0.001, ^****^ *p* < 0.0001. The ns indicates no significance.

To further assess the therapeutic effect of the Pt‐M@*B. sub* MN patch in treating diabetic infected wounds, tissue regeneration at the wound site was evaluated. Hematoxylin and eosin (H&E) staining revealed that the Pt‐M@*B. sub* MNs group exhibited a more complete skin architecture with significantly reduced inflammatory cell infiltration compared to other groups (**Figure** **9**a). Masson staining further demonstrated a notably larger area of collagen deposition in the regenerated epithelium following Pt‐M@*B. sub* MNs treatment, with a collagen volume fraction (CVF) of 65.22% ± 3.26%, markedly higher than other treatment groups (Figure 9b,e). To assess the local inflammatory response, IL‐6 and TNF‐α expression in wound tissues was measured. Immunohistochemical analysis indicated markedly lower IL‐6 levels in the Pt‐M@*B. sub* MNs group relative to the model group (Figure 9c,f), consistent outcomes were validated through TNF‐α immunohistochemical imaging and quantitative analysis (Figure S15a,c, Supporting Information), confirming the potent anti‐inflammatory effect of the treatment. Furthermore, CD86 immunofluorescence staining confirmed significantly reduced CD86 expression in wound tissues treated with Pt‐M@*B. sub* MNs, indicating decreased pro‐inflammatory M1 macrophage infiltration and attenuated inflammatory responses (Figure S15b,d, Supporting Information). Notably, compared to the model group, *B. subtilis* treatment did not induce inflammatory responses in skin tissue. Conversely, it significantly reduced levels of pro‐inflammatory cytokines TNF‐α and IL‐6 while alleviating expression of the M1 macrophage marker CD86. This anti‐inflammatory effect is likely attributable to reduced endotoxin secretion from *S. aureus* resulting from the probiotic's antibacterial clearance activity. Additionally, angiogenesis, a critical factor in diabetic wound repair, was evaluated by analyzing CD31 that is a well‐established marker of neovascularization. Immunofluorescence staining showed a significant upregulation of CD31 expression in the Pt‐M@*B. sub* MNs group, indicating enhanced vascular regeneration (Figure 9d,g).

Moreover, agar plate culture results confirmed that *B. subtilis* was nearly completely eliminated in vivo within 15 days (Figure S16, Supporting Information), demonstrating the favorable biosafety profile of the probiotic‐based treatment. H&E staining of major organs after Pt‐M@*B. sub* MNs treatment, including the heart, liver, spleen, lungs, and kidneys, revealed no observable pathological abnormalities (Figure S17, Supporting Information). Additionally, serum biochemical markers, including alanine aminotransferase (ALT), total bile acid (TBA), blood urea nitrogen (BUN), and creatinine (CR) remained within normal physiological ranges (Figure S18, Supporting Information), further supporting the systemic biocompatibility of the MN patch. Collectively, these findings confirm that the Pt‐M@*B. sub* MN patch not only exhibits outstanding biocompatibility but also promotes effective tissue regeneration, offering significant therapeutic potential for the treatment of diabetic infected wounds.

## 3 Discussion

In summary, we have developed a multifunctional probiotic MN patch incorporating platinum nanozyme‐modified *B. subtilis* (Pt‐M@*B. sub*) for the effective treatment of chronic diabetic wounds. The Pt‐M NZ modification significantly enhanced the survival and therapeutic performance of *B. subtilis* by scavenging ROS and generating oxygen in situ, thereby addressing two critical barriers of oxidative stress and hypoxia in diabetic wound healing. Embedded within a dissolvable, biocompatible polymer matrix, the MN patch enabled rapid and minimally invasive transdermal delivery of the therapeutic living material. In vivo results demonstrated that the Pt‐M@*B. sub* MN patch effectively exhibited robust antibacterial and anti‐inflammatory activity, promoted collagen deposition and angiogenesis, and accelerated wound closure. Furthermore, the MNs demonstrated efficient skin penetration and drug delivery in human cadaver skin, highlighting its clinical translation potential.

Pt‐based nanozymes, probiotics, and MNs have each been explored for promoting wound healing in previous studies. For example, PtTeCu nanosheets exhibited potent ROS‐scavenging and antibacterial properties in diabetic wound models.^[^ ^51^ ^]^ Probiotic‐loaded MNs, such as those delivering *Lactobacillus reuteri*, accelerated infected wound healing via the secretion of reuterin, a natural antimicrobial agent.^[^ ^32^ ^]^ Similarly, MNs encapsulating *Chlorella* have been used to overcome biological barriers, offering both antibacterial effects and sustained oxygen release at wound sites.^[^ ^52^, ^53^ ^]^ While these individual strategies show promise, they each face limitations—Pt‐based nanozymes often overlook the challenge posed by biofilm barriers and may damage commensal microbiota, whereas probiotic‐based MN systems mainly rely on metabolite production or oxygen supply, without addressing oxidative stress in a comprehensive manner. In this study, we developed a synergistic therapeutic platform that overcomes these limitations by integrating Pt‐based nanozymes directly onto the surface of *B. subtilis*, and further embedding the nanozyme‐engineered probiotics within dissolving MNs. This design offers three key synergistic mechanisms: (1) MNs physically disrupt pathogenic biofilms, enabling deep and localized delivery; (2) Pt‐M NZ effectively scavenge ROS and catalytically generate oxygen in situ, reducing oxidative damage and supporting a more favorable wound environment; (3) the locally generated oxygen enhances the viability and metabolic activity of *B. subtilis*, sustaining its antimicrobial effects for over 12 days. Nevertheless, the long‐term efficacy, stability, and biosafety of the probiotic‐based MN system require further evaluation.

Although metabolites derived from *B. subtilis* or conventional antibiotics can inhibit pathogenic bacteria, their effects are transient and require repeated administration to maintain efficacy. In contrast, Pt‐M@*B. sub* enabled the prolonged survival of *B. subtilis* at the wound site for up to 12 days, thereby ensuring sustained antibacterial activity without the need for multiple doses. Importantly, the use of Pt‐M@*B. sub* mitigates the risks associated with antibiotic overuse and repeated exposure, which are well known to drive resistance. Furthermore, emerging evidence demonstrates that *B. subtilis* not only suppresses pathogens but also modulates host immune responses, attenuates pro‐inflammatory cytokines, and promotes tissue repair,^[^ ^54^ ^]^ which cannot be achieved by antibiotics or single metabolites. Therefore, Pt‐M@*B. sub* represents a dynamic, self‐sustaining, and host‐beneficial living therapeutic uniquely suited for the treatment of infected wounds.

Collectively, this work presents a synergistic therapeutic platform that combines living probiotics and catalytic nanomaterials in a MN‐based delivery system, offering a promising strategy for the treatment of infected diabetic wounds and advancing the broader application of living engineered materials in regenerative medicine.

## 4 Experimental Section

### Materials

Potassium tetrachloroplatinate (K_2_PtCl_4_) was purchased from Bidepharm (Shanghai, China). L‐ascorbic acid (LAA), 2,2‐Diphenyl‐1‐picrylhydrazyl (DPPH), Salicylic Acid, Ferrous Sulfate Heptahydrate and sucrose were purchased from Macklin (Shanghai, China). Polyoxyethylene 20 cetyl ether (Brij 58), 4‐Mercaptophenylboronic acid (4‐MPBA), hydrogen peroxide (H_2_O_2_), nitroblue tetrazolium chloride (NBT), riboflavin, 2,2′‐azino‐bis (3‐ethylbenzothiazoline‐6‐sulfonic acid) (ABTS), [Ru(dpp)_3_]Cl_2_ and streptozotocin (STZ) were purchased from Aladdin (Shanghai, China). Polyvinyl alcohol (PVA) (Mw 9000 – 10 000) and Polystyrene (PS) (Mw ∼ 192 000) were purchased from Sigma‐Aldrich (USA). Antibodies against TNF‐α, IL‐6, and HIF‐1α were purchased from MCE (USA). Dulbecco's Modified Eagle's Medium (DMEM), fetal bovine serum (FBS), and penicillin‐streptomycin double antibiotics were purchased from Gibco (USA). Phosphate‐buffered saline (PBS) was purchased from Servicebio (Wuhan, China). Cell Counting Kit‐8 (CCK‐8) and 4% paraformaldehyde fix solution were purchased from Biosharp (Hefei, China). Calcein/PI Cell Viability/Cytotoxicity Assay Kit was purchased from Beyotime (Shanghai, China). Reactive Oxygen Species Assay Kit, CAT Activity Assay Kit (Ammonium Molybdate‐Chromogenic Method), SOD Activity Assay Kit (WST‐1 Method), and Gram staining kit were purchased from Solarbio (Beijing, China). 25% Glutaric dialdehyde and triton X‐100 were purchased from Sinopharm (Shanghai, China). Trypticase Soy Broth (TSB), Tryptone Soy Agar (TSA), 1% dipotassium trioxotellurate solution, and Desoxycholate Lactose Agar (DC) were purchased from Hopebio (Qingdao, China). Polydimethylsiloxane (PDMS) was purchased from Dow Corning (Mid‐land, USA).

### Bacteria and Cell Lines

*B. subtilis* (ATCC 6633) was purchased from the Shanghai Microbiological Culture Collection (Shanghai, China) and cultured in TSB nutrient broth. The *S. aureus* and *E. coli* were obtained from the American Type Culture Collection (ATCC, Rockville, MD, USA) and cultured in TSB nutrient broth. HaCaT (RRID: CVCL_0038), NIH‐3T3 (RRID: CVCL_0594) cell lines, and HUVECs (RRID: CVCL_2959) were purchased from Procell Biological Co., LTD (Wuhan, China). It was confirmed that the cell lines were free of contamination at the time of use. The cells were cultured in DMEM High Glucose supplemented with 10% FBS and 1% penicillin‐streptomycin at 37 °C with 5% CO_2_.

### Synthesis of Pt NZ and Pt‐M NZ

A 10 mL K_2_PtCl_4_ (10 mm) and Brij 58 (4.45 mm) aqueous solution was mixed with 10 mL L‐ascorbic acid (50 mm) aqueous solution under ultrasonication for 45 min. The color change of the solution from orange‐yellow to black indicated the successful synthesis of Pt NZ. The Pt NZ was collected by centrifuged at 11 000 rpm for 20 min. To synthesize Pt‐M NZ, the Pt NZ solution was mixed with 20 mL of an ethanol solution containing 0.5 mg mL^−1^ 4‐mercaptophenylboronic acid (4‐MPBA) and stirred for 1 h. The mixture was centrifuged at 11000 rpm for 20 min, washed three times with ethanol, and dispersed in 20 mL deionized (DI) water to obtain Pt‐M NZ. The particle size and zeta potential of Pt NZ and Pt‐M NZ were detected by DLS (Malvern, Zetasizer Nano ZSP), and their morphology was characterized by TEM (JEOL, JEM‐F200). The platinum (Pt) content in Pt‐M NZ was determined by Inductively coupled plasma mass spectrometry (ICP‐MS) (Analytik‐Jena, PQ‐MS).

### SOD and CAT like Activity of Pt‐M NZ

The SOD‐like activity of Pt‐M NZ was evaluated by using nitroblue tetrazolium chloride (NBT) as the substrate. Riboflavin generates •O_2_^−^ under light, which reduces NBT to blue formazan. A decrease in blue formazan formation indicates stronger SOD‐like activity. In order to assess the SOD‐like activity of Pt‐M NZ, riboflavin and NBT were incubated with different concentrations of Pt‐M NZ under light for 20 min, and then the ultraviolet absorption spectrum of the solution was scanned by UV–vis spectrophotometer (Aoe, Aoe A590). The CAT‐like activity of Pt‐M NZ was assessed by measuring oxygen generation during the decomposition of H_2_O_2_. Specifically, a 10 mm H_2_O_2_ solution was incubated with different concentrations of Pt‐M NZ, and the dissolved oxygen content in the solution was monitored at various time points using a portable dissolved oxygen meter.

### ABTS Radical Scavenging Activity

First, 7.4 mm ABTS diammonium salt solution and 2.6 mm potassium persulfate were mixed and reacted in the dark for 12 h to obtain ABTS free radical stock solution, and then the ABTS stock solution was diluted with 1 × PBS buffer to obtain ABTS free radical working solution. After incubating the ABTS working solution with different concentrations of Pt‐M NZ in the dark for 30 min, the ultraviolet absorption spectrum of the solution was scanned by a UV–vis spectrophotometer. The ABTS radical scavenging rate was calculated based on the absorbance value of the solution at 734 nm, and the ABTS scavenging rate (%) was calculated as follows: ABTS scavenging rate (%) = (A_0_‐A_c_)/A_0_, while A_0_ is the absorbance of ABTS solution at 734 nm without any treatment, and A_c_ is the absorbance of ABTS solution at 734 nm after treatments with different concentrations of Pt‐M NZ.

### DPPH Radical Scavenging Activity

Initially, 5 mg DPPH was dissolved in 100 mL absolute ethanol to form DPPH free radical working solution. After incubating the DPPH working solution with different concentrations of Pt‐M NZ in the dark for 30 min, the ultraviolet absorption spectrum of the solution was scanned by a UV–vis spectrophotometer. The DPPH radical scavenging rate was calculated based on the absorbance value of the solution at 517 nm, the DPPH scavenging rate (%) was calculated as follows: DPPH scavenging rate (%) = (A_0_‐A_c_)/A_0_, where A_0_ is the absorbance of DPPH solution at 517 nm without any treatment, and A_c_ is the absorbance of DPPH solution at 517 nm after treatments with different concentrations of Pt‐M NZ.

### Assessment of Intracellular ROS Levels

Intracellular ROS levels in HaCaT cells and NIH‐3T3 cells were measured using the fluorescent probe DCFH‐DA. HaCaT cells and NIH‐3T3 cells were cultured in DMEM High Glucose supplemented with 10% FBS and 1% penicillin‐streptomycin at 37 °C with 5% CO_2_. Briefly, cells were seeded in 6‐well plates and cultured for 24 h, and then the cells in H_2_O_2_ + Pt‐M NZ group were given 30 µg mL^−1^ Pt‐M NZ pretreatment overnight. Subsequently, cells in H_2_O_2_ group and H_2_O_2_ + Pt‐M NZ group were stimulated with 400 µm H_2_O_2_ for 6 h. Cell samples were washed by PBS and then incubated with DCFH‐DA probe for 15 min, and washed three times with PBS. Intracellular ROS levels were evaluated by an inverted fluorescence microscope and flow cytometry.

### Intracellular SOD and CAT Activity Assays

HaCaT cells and NIH‐3T3 were cultured in DMEM High Glucose supplemented with 10% FBS and 1% penicillin‐streptomycin at 37 °C with 5% CO_2_. The cells were seeded in cell culture dishes and cultured for 24 h. The cells in H_2_O_2_ + Pt‐M NZ group were pretreated with 30 µg mL^−1^ Pt‐M NZ overnight. Subsequently, cells in the H_2_O_2_ group and the H_2_O_2_ + Pt‐M NZ group were stimulated with 400 µm H_2_O_2_ for 6 h. Intracellular SOD and CAT activities were measured using commercial CAT and SOD detection kits.

### Evaluation of the Anti‐Inflammatory Effect

RAW264.7 cells were seeded in culture dishes and incubated overnight, followed by stimulation with 100 ng mL^−1^ LPS for 12 h to induce M1 polarization. Subsequently, the RAW264.7 cells were cocultured with 30 µg mL^−1^ Pt‐M NZ for 12 h. Cells were labeled with CD86‐APC and CD206‐PE and detected by flow cytometry to analyze the proportion of M1 macrophages. Pro‐inflammatory cytokines, including TNF‐α and IL‐6, were detected by real‐time quantitative polymerase chain reaction (RT‐qPCR). For immunofluorescence experiments, macrophage samples were fixed with 4% paraformaldehyde, permeabilized with Triton X‐100, and blocked with 1% BSA. Samples were incubated overnight with TNF‐α or IL‐6 antibodies at 4 °C, followed by secondary antibodies and DAPI staining. Finally, the samples were mounted with the antifluorescence quencherizer and observed and recorded under the inverted fluorescence microscope.

### Preparation and Characterization of Pt‐M@*B. sub*

The prepared Pt‐M NZ (400 µg mL^−1^) was incubated with *B. subtilis* (1×10^9^ CFU mL^−1^) in PBS solution for 4 h, followed by centrifugation at 4500 rpm for 10 min to obtain Pt‐M@*B. sub*. The conjugation efficiency was determined by measuring the platinum concentration in both the original Pt‐M NZ solution and the supernatant (containing unbound Pt‐M NZ) using ICP‐MS. Conjugation Efficiency (%) = (1 – Concentration of unconjugated Pt / Initial concentration of Pt) × 100. The morphology of *B. subtilis* and Pt‐M@*B. sub* were observed by Field Emission Scanning Electron Microscope (FESEM) (Tescan, Tescan MIRA).

### Impact Evaluation of Pt‐M NZ Modification on *B. subtilis* Viability

*B. subtilis* cultures were divided equally into two groups: (1) free probiotic suspended in PBS, and (2) Pt‐M NZ‐conjugated probiotic (Pt‐M@*B. sub*). The latter was prepared by conjugating *B. subtilis* with Pt‐M NZ followed by centrifugation. Both groups were incubated in PBS for 24 h at 37 °C. Subsequently, aliquots from each bacterial suspension were spread‐plated on TSA plates and incubated at 37 °C for 24 h. Following photographic documentation, colony‐forming units were quantified to assess the impact of Pt‐M NZ conjugation on bacterial viability.

### The Protective Effect of Pt‐M@*B. sub* on *B. subtilis* Viability under High‐ROS/hypoxic Conditions

The Control group comprised *B. subtilis* suspended in PBS under normoxic conditions; the hypoxia control (Blank) group consisted of anaerobically treated *B. subtilis* in an anaerobic pouch (0.1% O_2_); while experimental groups included H_2_O_2_ (ROS/hypoxia‐treated *B. subtilis*), H_2_O_2_ + Pt NZ (physical mixture of Pt NZ and *B. subtilis* under ROS/hypoxia), and H_2_O_2_ + Pt‐M NZ (Pt‐M@*B. sub* under ROS/hypoxia), all groups were incubated for 24 h before SEM sample preparation involving ethanol gradient dehydration, silicon wafer deposition, gold‐sputtering, and imaging. For viability assessment, treated bacterial suspensions were spread‐plated on TSA plates, incubated at 37 °C for 24 h, photographed, and colony‐forming units quantified.

### The Hypoxia‐Alleviating Capacity of Pt‐M@*B. sub*

The hypoxia‐alleviating capacity of Pt‐M@*B. sub* was evaluated through intracellular oxygen detection using [Ru(dpp)_3_]Cl_2_ probe and HIF‐1α immunofluorescence staining. For oxygen measurement, HaCaT or NIH‐3T3 cells were seeded in 6‐well plates overnight for adhesion, subjected to 4 h hypoxia in an anaerobic pouch (0.1% O_2_, 5% CO_2_), then treated with H_2_O_2_, H_2_O_2_ + Pt‐M NZ, or H_2_O_2_ + Pt‐M@*B. sub*‐containing medium under continued hypoxia for 6 h, followed by 30‐min co‐staining with Hoechst 33 342 and [Ru(dpp)_3_]Cl_2_ prior to inverted fluorescence microscopy imaging. For HIF‐1α detection, similarly plated cells underwent 6 h hypoxia (0.1% O_2_, 5% CO_2_), received identical treatments during 8 h extended hypoxia, then were fixed with 4% PFA, permeabilized with 0.1% Triton X‐100, blocked with 1% BSA (1 h, RT), incubated with HIF‐1α antibody overnight (4 °C), stained with secondary antibody (1 h, RT, dark), counterstained with DAPI (30 min, RT, dark), mounted with antifade medium, and finally imaged using inverted fluorescence microscopy.

### Tube Formation Assay

Matrigel was added to 24‐well plates and polymerized at 37 °C for 1 h. HUVECs were then seeded onto the gel‐coated wells and treated with experimental groups (Control, H_2_O_2_, H_2_O_2_ + Pt‐M NZ, H_2_O_2_ + Pt‐M@*B. sub*). After 6 h incubation, tube formation was imaged using phase‐contrast inverted microscopy and total tube length quantified with ImageJ software.

### Cell Migration

HaCaT cells were seeded in 6‐well plates and cultured in DMEM supplemented with 1% FBS and 1% penicillin‐streptomycin until reaching approximately 80% confluency (Cells were treated with 1% FBS to minimize interference from cell proliferation on the migration assay results). The cells were treated with different treatment groups (Control, H_2_O_2_, H_2_O_2_ + Pt‐M NZ and H_2_O_2_ + Pt‐M@*B. sub*). The concentrations were set as follows: H_2_O_2_ at 400 µm, Pt‐M NZ at 30 µg mL^−1^, and Pt‐M@*B. sub* (containing 30 µg mL^−1^ Pt‐M NZ and 8.3 × 10⁷ CFU/mL *B. subtilis*). Cell scratches were caused by scratching the cells with a pipette, and images of cell migration at 0, 12, 24, 48 h were monitored by the inverted fluorescence microscope.

### Antibacterial Properties of Pt‐M@*B. sub* in the Condition of H_2_O_2_ and Hypoxia

The antibacterial activity of Pt‐M@*B. sub* in the condition of H_2_O_2_ and hypoxia was evaluated using *S. aureus* and *E. coli*. Different therapeutic agents (Control, *B. subtilis*, Pt/*B. sub*, Pt‐M@*B. sub*) were added to bacterial culture tubes containing 200 µM H_2_O_2_ and 1×10^6^ CFU mL^−1^ *S. aureus*, followed by incubation in anaerobic culture bags (0.1% O_2_). The concentrations were configured as follows: the *B. subtilis* group contained 8.3 × 10⁷ CFU/mL *B. subtilis*, the Pt/*B. sub* group contained 30 µg mL^−1^ Pt NZ and 8.3 × 10⁷ CFU/mL *B. subtilis*, and the Pt‐M@*B. sub* group contained 30 µg mL^−1^ Pt‐M NZ and 8.3 × 10⁷ CFU/mL *B. subtilis*. After incubation for 24 h, 100 µL of suspensions were plated on TSA‐DT agar and cultured for 48 h for colony quantification. Similarly, *E. coli* was co‐cultured with therapeutic agents, diluted 10^4^‐fold, plated on DC agar, and incubated for 24 h for colony enumeration.

### Fabrication and Characterization of Pt‐M@*B. sub* MN Patch

The Pt‐M@*B. sub* MN patch was fabricated using polydimethylsiloxane (PDMS) molds (Dow Corning, Midland, USA). A Pt‐M@*B. sub* suspension, containing 1.08 mg mL^−1^ Pt‐M NZ and 3 × 10^9^ CFU mL^−1^ *B. subtilis*, was incorporated into a matrix solution composed of 18% (w/v) PVA and 18% (w/v) sucrose, then 100 µL of the matrix solution was pipetted dropwise on the surface of the PDMS mold, vacuumed at a level of 0.1 MPa for 4 h. The concentrations of Pt‐M NZ and *B. subtilis* were selected based on relevant literature.^[^ ^27^, ^55^ ^]^ After that, 60 µL of 20% (w/v) PS matrix solution was added dropwise, followed by another 4 h of vacuum treatment to form the backing layer. The mold was then dried at room temperature for 2 days, after which the MN patch was carefully peeled off and stored in a desiccator at 4 °C until use. The morphology of Pt‐M@*B. sub* MN patch was observed by optical microscope and SEM, and its mechanical strength was measured by the mechanical tester.

### Penetration Assessment of Pt‐M@*B. sub* MN Patch in Porcine Skin and Human Cadaver Skin *Ex Vivo*

The skin‐penetrating ability of the MN patch was evaluated by being inserted into fresh porcine skin or human cadaver skin for 5 min. All human skin experiments were approved by the Ethics Committee of Wuhan Third Hospital, Wuhan University (approval number: KY2025‐029). Frozen section of human skin tissue after Pt‐M@*B. sub* MN patch penetration was observed by an inverted microscope (OLYMPUS, IX73). Gram staining kit was used to staining the human skin tissue to confirm the successful delivery of Pt‐M@*B. sub*.

### Cytotoxicity Assay In Vitro

HaCaT cells and NIH‐3T3 cells were used for evaluating the cytotoxicity of Pt‐M NZ and *B. subtilis*. Briefly, cells were seeded into 96‐well plates, cultured in DMEM medium containing 10% FBS and 1% penicillin‐streptomycin solution for 24 h, and then different concentrations of Pt‐M NZ or *B. subtilis* (ultrasonic crushing followed by lyophilization) were co‐incubated with cells for 24 h. Cell viability was assessed using the CCK‐8 assay, with absorbance measured at 450 nm using a microplate reader. In order to verify the biosafety of the MN patches, the cells were co‐incubated with different types of MN patches for 24 h, stained with calcein‐AM/PI, and imaged under an inverted fluorescence microscope to distinguish live and dead cells.

### Hemolysis Experiment

The biocompatibility of the MN patches was detected by the hemolysis test. Blood (2 mL) was collected from rats via orbital venous puncture and transferred to heparin sodium‐coated tubes. The blood was centrifuged at 1500 rpm for 10 min, and the supernatant was discarded. The erythrocyte pellet was resuspended in 6 mL saline to prepare the erythrocyte suspension. Different MN patches were dissolved in 1 mL of saline to prepare test solutions. A 200 µL aliquot of the erythrocyte suspension was incubated overnight with the test solutions, saline (negative control), or deionized water (positive control). After centrifugation at 1500 rpm for 10 min, the absorbance of the supernatant was measured at 540 nm. The hemolysis rate was calculated using the following formula: Hemolysis rate (%) = (A_test_ – A_negative_) / (A_positive –_ A_negative_), where A_test_ is the absorbance value of different types of test solutions, A_negative_ is the absorbance value of the negative control group, and A_positive_ is the absorbance value of the positive control group.

### Application of Pt‐M@*B. sub* MN Patch for Diabetic Infection Wound Treatment In Vivo

All animal experiments were conducted in accordance with protocols approved by the Institutional Animal Care and Use Committee of Wuhan University (approval number: 20 241 135). Male Sprague‐Dawley (SD) rats were used as experimental animals in this study, which were purchased from China Three Gorges University with approval number SCXK 2020‐0005. The rats were housed under a 12 + 12 h dark/light cycle, and subjected to a controlled ambient temperature of 20–25 °C, and the rats had access to adequate food and water during the experiment. Rats were induced into the diabetic model by injecting streptozotocin (35 mg kg^−1^) for three consecutive days. The blood glucose of rats was monitored by a blood glucose meter, and when the blood glucose of rats was higher than 16.7 mmol L^−1^ for one consecutive week, it proved that the diabetes model was successfully induced. Then, a full‐thickness skin wound with a diameter of 1 cm was created on the dorsal skin of the rats, followed by inoculation with *S. aureus* (100 µL, 10^8^ CFU mL^−1^) at the wound site to establish a diabetic infected wound model. The rats were randomly divided into seven groups (n = 5): (1) No treatment (model group), (2) Blank MNs, (3) Pt‐M MNs, (4) *B. sub* MNs, (5) Pt/B. *sub* MNs, (6) Pt‐M@B. *sub* MNs and (7) Pt‐M@*B. sub* Hydrogel. Wound images were captured on days 0, 3, 5, 8, 12, and 15. Wound healing rates were calculated as: Wound healing rate (%) = [(original wound area – actual wound area)/original wound area] × 100%. In addition, rat blood glucose was monitored by the blood glucose meter throughout the experiment. Moreover, the wound skin of rats in different MN treatment groups were homogenized in 10 mL sterile PBS solution on day 5. Then, 100 µL of the bacterial suspension were seeded on TSA‐DT agar medium, and the number of colonies was calculated after 48 h to evaluate the antibacterial efficacy of the MN patch in vivo. Finally, the dorsal wound skin of the rats was cut off on day 15 for H&E staining, Masson staining, or immunofluorescence staining.

### Biosafety of Pt‐M@*B. sub* MN Patches In Vivo

Different types of MN patches were applied to the dorsal skin of rats. After 15 days, plasma samples were collected, and liver (ALT, TBA) and kidney (BUN, CR) function markers were analyzed using a BK‐280 automatic biochemical analyzer. Additionally, major organs (heart, liver, spleen, lung, and kidney) were harvested for H&E staining and histological analysis.

### Statistical Analysis

All results of this study were exhibited as mean ± standard deviation (SD), and analyzed by the software GraphPad Prism 8.0. The statistical analysis was evaluated by using a one‐way analysis of variance (ANOVA). The value *p* < 0.05 was recognized as statistically significant. The levels of significance were labeled as ^*^ *p* < 0.05, ^**^ *p* < 0.01, ^***^ *p* < 0.001, and ^****^ *p* < 0.0001.

## Conflict of Interest

The authors declare no conflict of interest.

## Supporting information



Supporting Information

## Data Availability

The data that support the findings of this study are available from the corresponding author upon reasonable request.
