# Supplementary material for: Nanozyme‐Engineered Probiotic Microneedle Patch for Chronic Diabetic Wound Therapy
Source: Adv Sci (Weinh). 2025 Sep 17;12(45):e12127. doi: 10.1002/advs.202512127 (PMC12677689; doi:10.1002/advs.202512127)
Supplement: Supplementary file 1 — Supporting Information [file ADVS-12-e12127-s001.docx]

**Supporting Information**


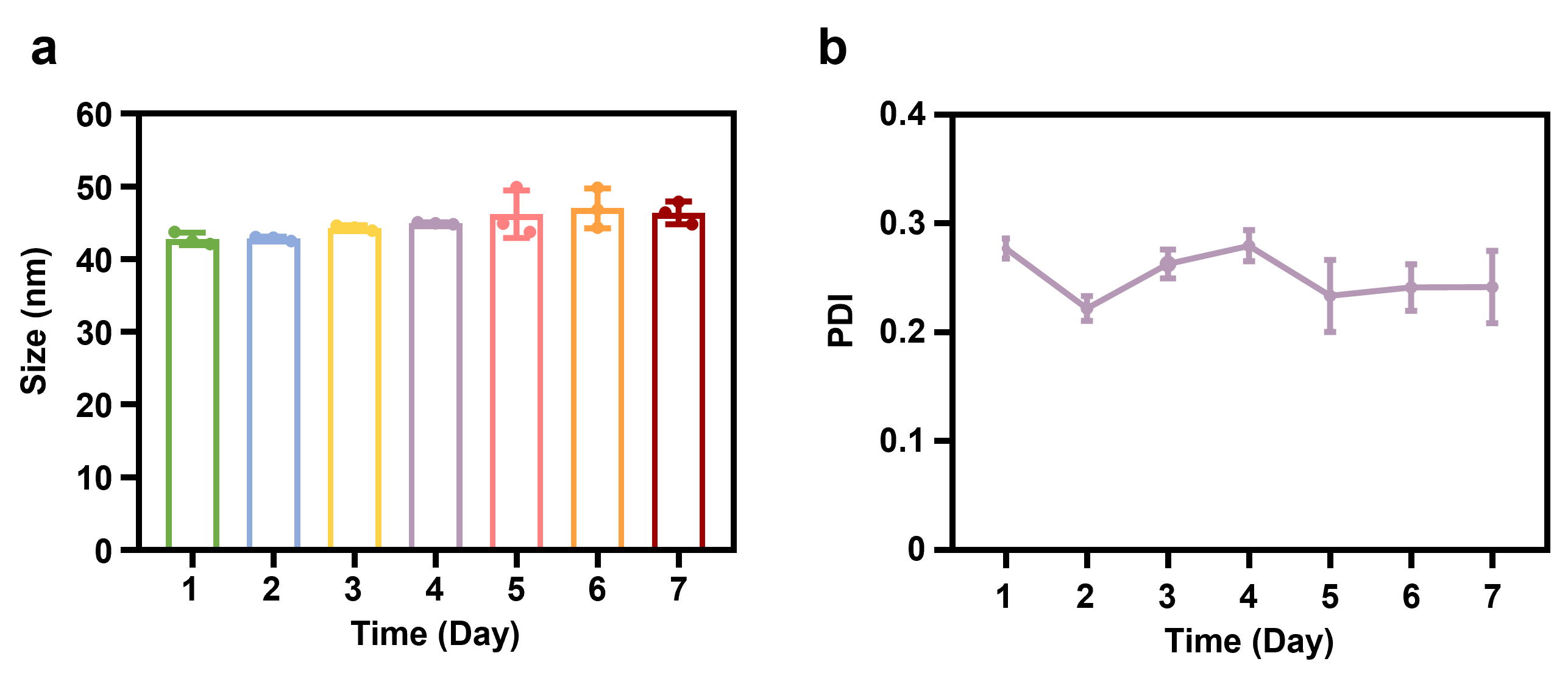
 **Figure S1.** Long-term stability of Pt-M NZ particles. **(a)** Particle size of Pt-M NZ at different time points. **(b)** Polydispersity index (PDI) of Pt-M NZ at different time points.


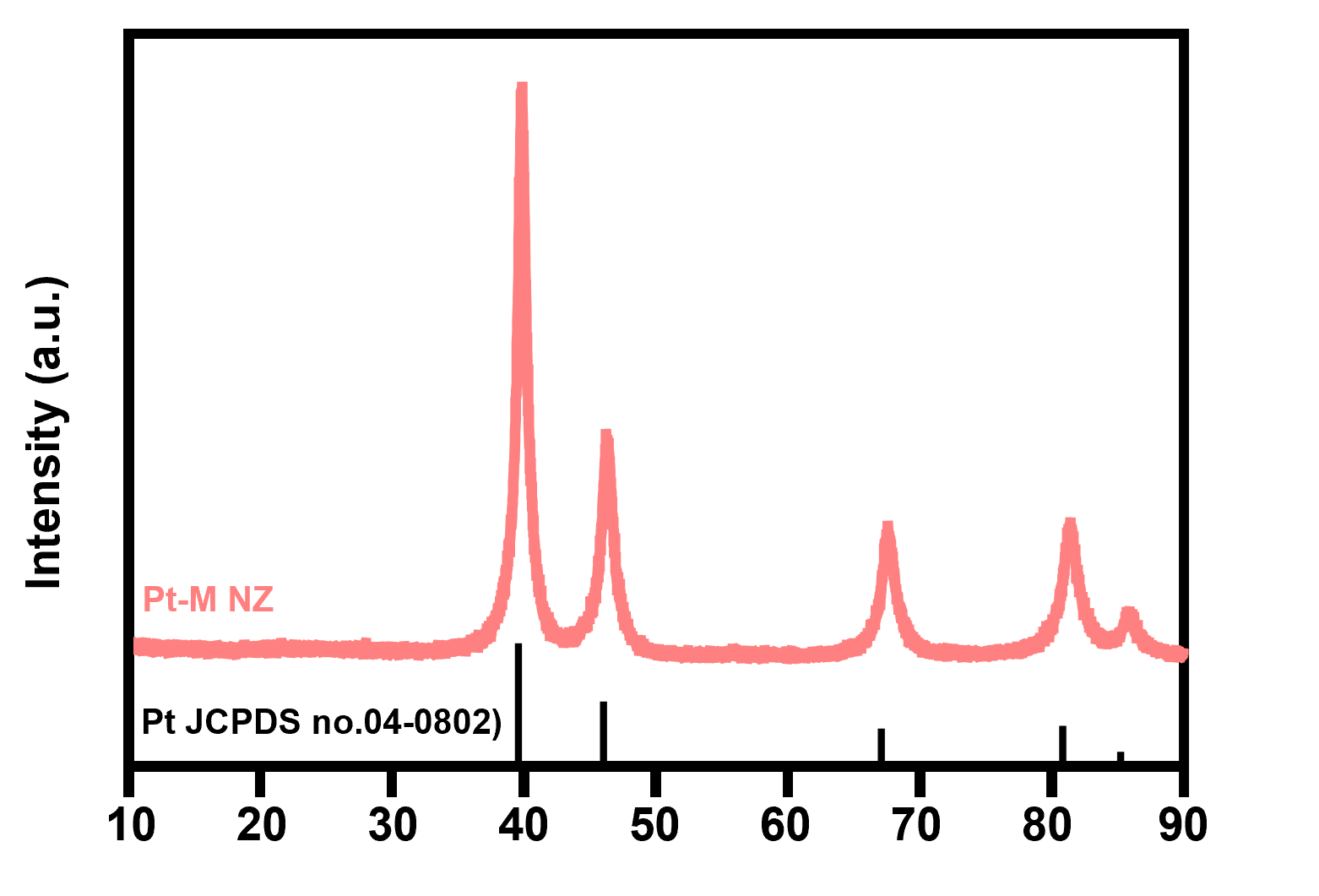


**Figure S2.** X-ray diffraction (XRD) pattern of Pt-M NZ.

**
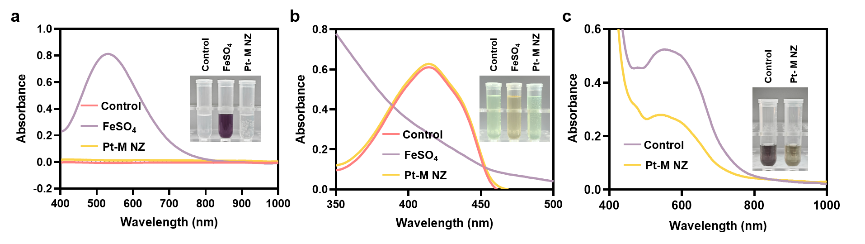
**

**Figure S3. (a)** UV-vis absorption spectra and color changes for ·OH generation detected using salicylic acid as the trapping agent in different treatment groups. **(b)** UV-vis absorption spectra and color changes for ·OH generation detected using DPBF as the trapping agent in different treatment groups. **(c)** UV-vis absorption spectra and color changes of NBT solution in different treatment groups during the •O_2_^-^ scavenging experiment.


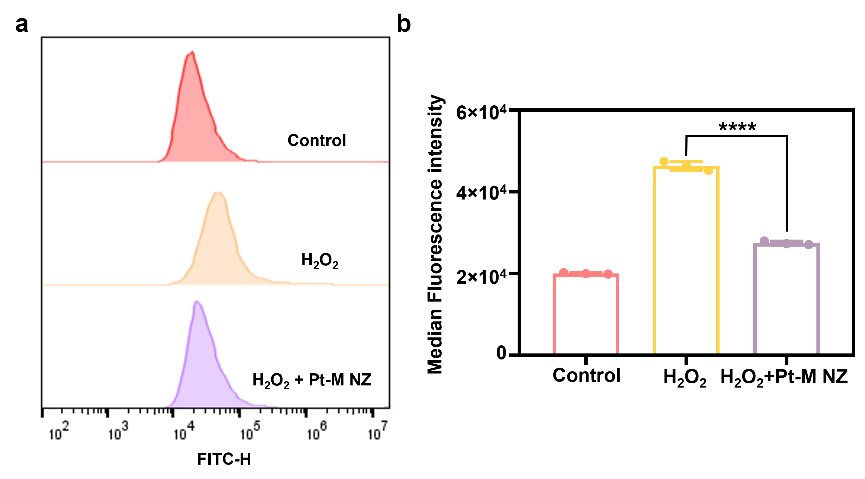


**Figure S4. (a)** Flow cytometry results and **(b)** quantitative statistics of NIH-3T3 cells stained with DCFH-DA to reflect the intracellular ROS level with different treatments. Each point represents mean ± SD (n = 3). *****P* < 0.0001.


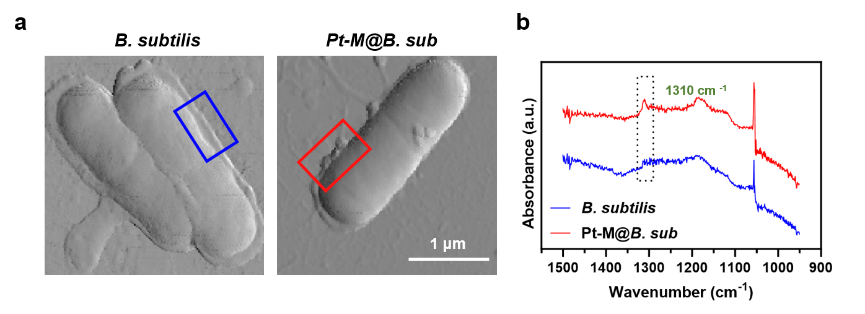


**Figure S5. (a)** Atomic force microscopy (AFM) images and **(b)** corresponding FTIR spectra of *B. subtilis* and Pt-M@*B. sub*.


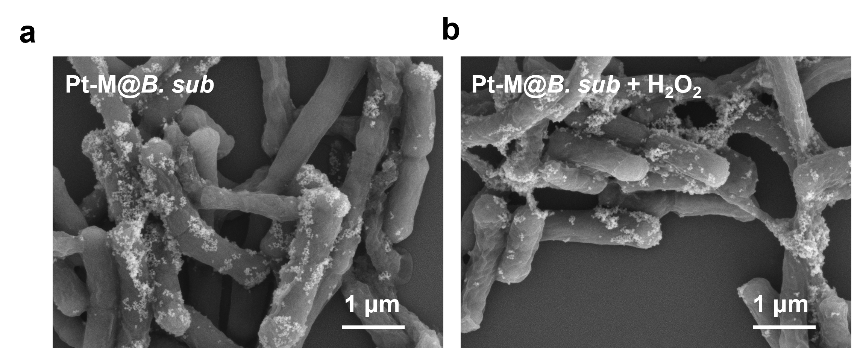


**Figure S6.** The SEM images of Pt-M@*B. sub* at the presence of 200 μM H_2_O_2_ for 24 h_._


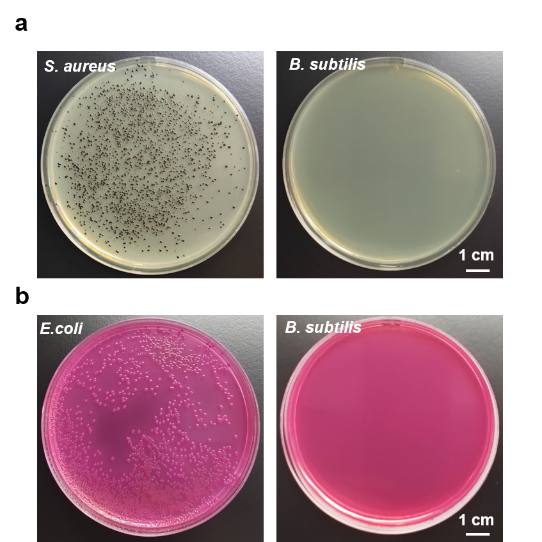


**Figure S7.** Representative photographs of **(a)** *S. aureus* and *B. subtilis* colonies on TSA-DT plate. Representative photographs of **(b)** *E. coli* and *B. subtilis* colonies on DC plate.

**
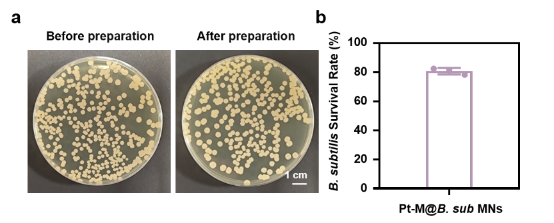
**

**Figure S8.** Plate coating images (**a**) and survival analysis (**b**) of *B. subtilis* before and after Pt-M@*B. sub* MN patch preparation.


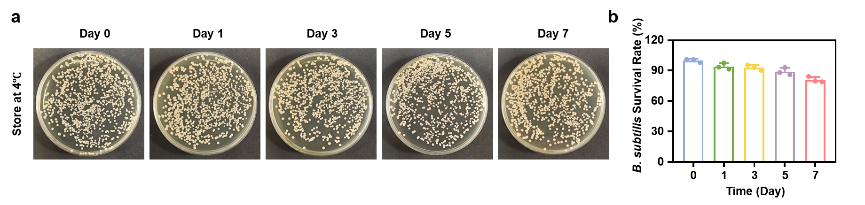


**Figure S9.** (**a**) Photographs and (**b**) survival viability of *B. subtilis* colonies on agar plates after storing Pt-M@*B. sub* MN at 4°C over time. Each point represents mean ± SD. (n = 3).

**
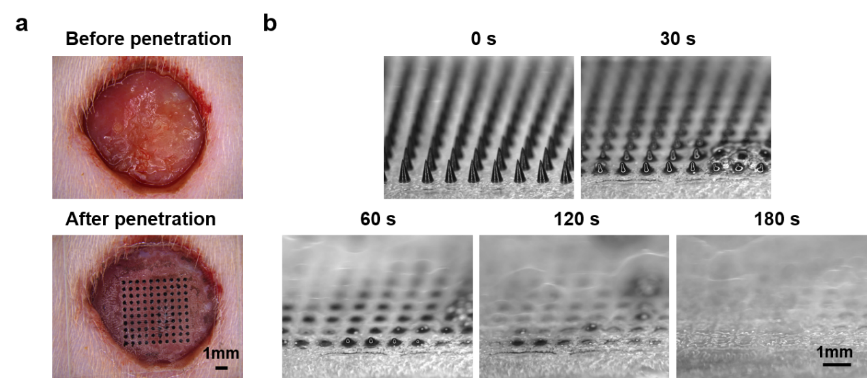
**

**Figure S10.** Evaluation of drug release from Pt-M@*B. sub* MN in diabetic infected wounds. **(a)** Images showing MNs administration at the wound site. **(b)** Dissolution images of MNs at different time points post-application.

**
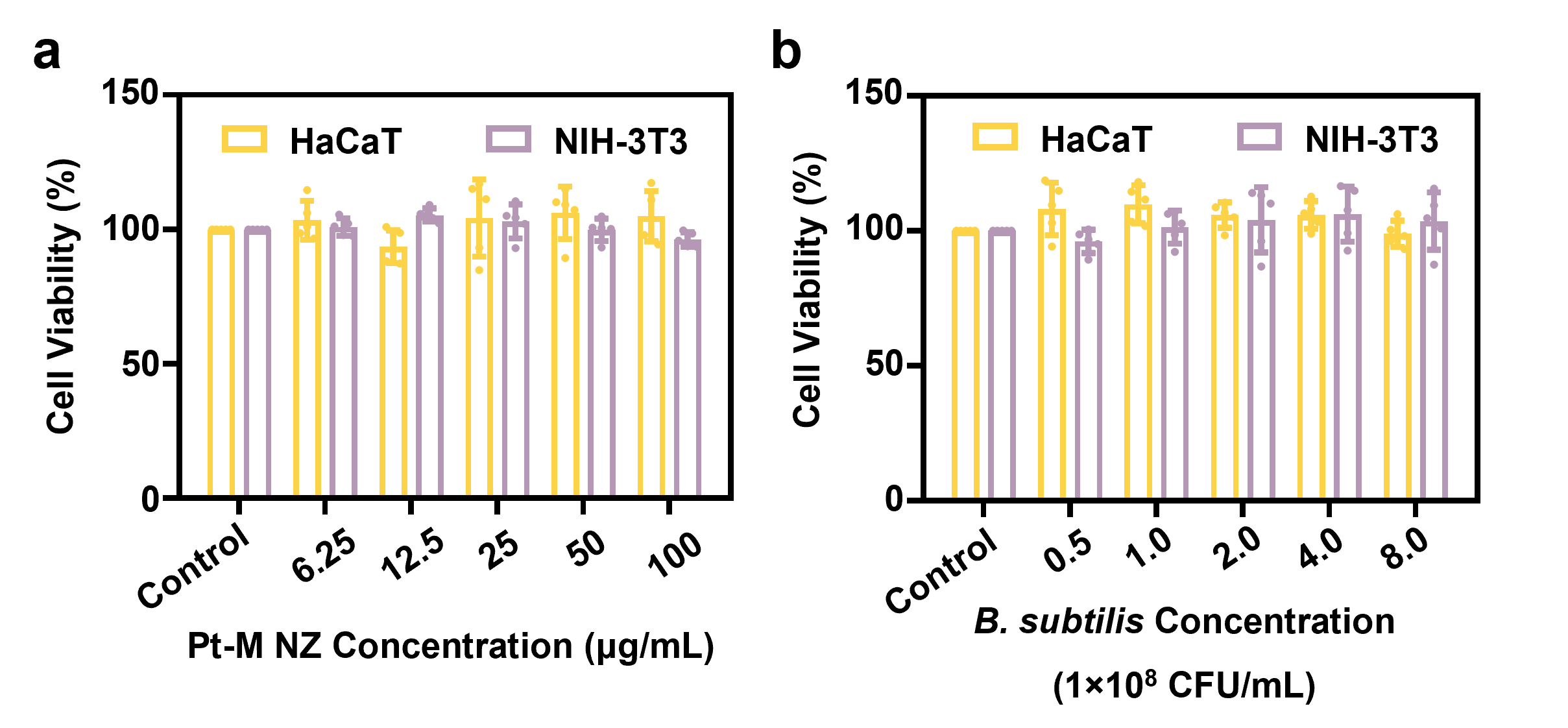
**

**Figure S11.** Cell viability analysis of HaCaT cells and NIH-3T3 cells after treatments with different concentrations of **(a)** Pt-M NZ and **(b)** *B. subtilis*. Each point represents mean ± SD (n = 5).

**
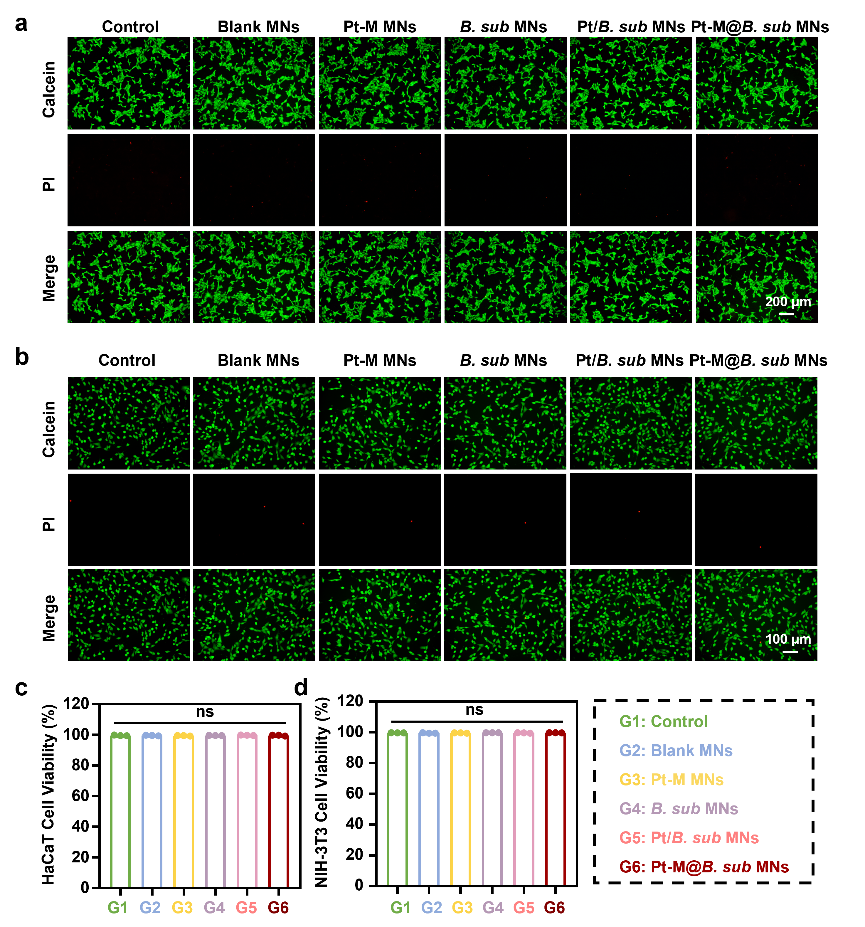
**

**Figure S12.** Live/dead cell fluorescence staining images of (**a**) HaCaT and (**b**) NIH-3T3 cells after receiving different MN treatments. Cell viability analysis of (**c**) HaCaT and (**d**) NIH-3T3 cells after receiving different MN treatments. Each point represents mean ± SD (n = 3). The ns indicates no significance.

**
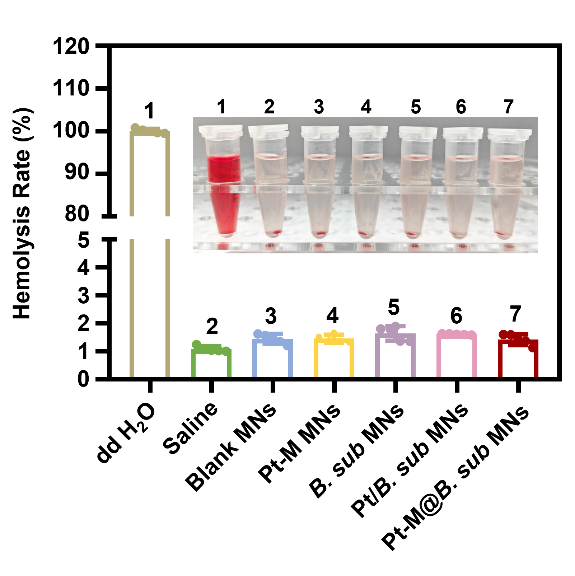
**

**Figure S13.** Hemolysis rate with different MN treatments. Each point represents mean ± SD (n = 5).


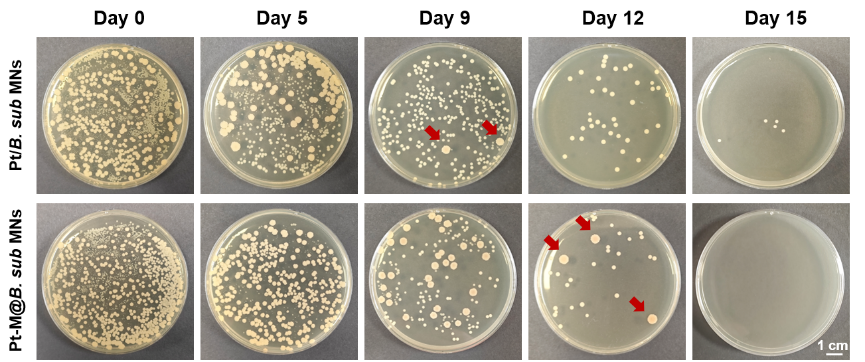


**Figure S14.** Results of cultured *B. subtilis* from diabetic infected wounds applied with Pt/*B. sub* MNs and Pt-M@*B. sub* MNs at different time points (The red arrows represent colonies of *B. subtilis*).

**
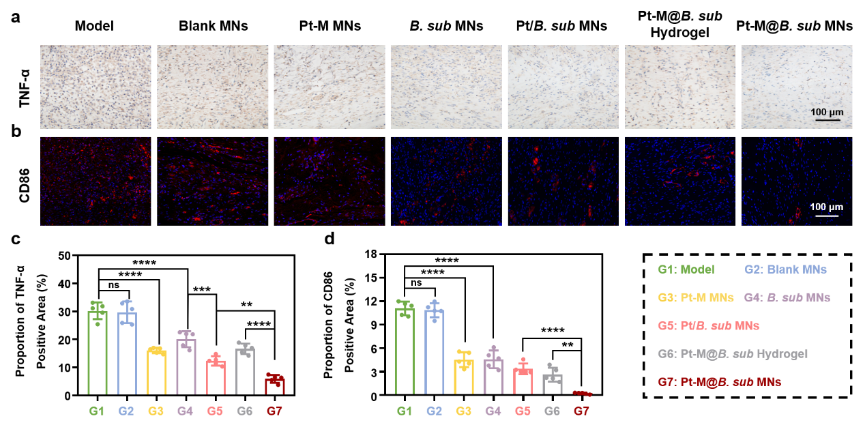
**

**Figure S15.** Histopathological analysis of diabetic infected wounds on day 15 post-treatment. **(a)** Immunohistochemistry staining of TNF-α after different treatments. **(b)** Representative immunofluorescence microscopy images of CD86 after different treatments. **(c)** Quantitative statistics of the positive areas for TNF-α immunohistochemical staining. **(d)** Quantitative statistics of the positive areas for CD86 immunofluorescence staining.


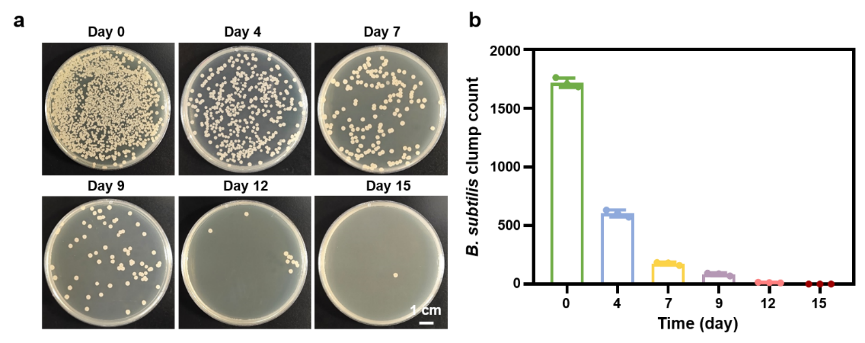


**Figure S16.** (**a**) Photographs of *B. subtilis* growth on agar plates after being extracted from rats after subcutaneous injection of *B. subtilis*. (**b**) The CFU of *B. subtilis* growth on agar plates. Each point represents mean ± SD (n = 3).


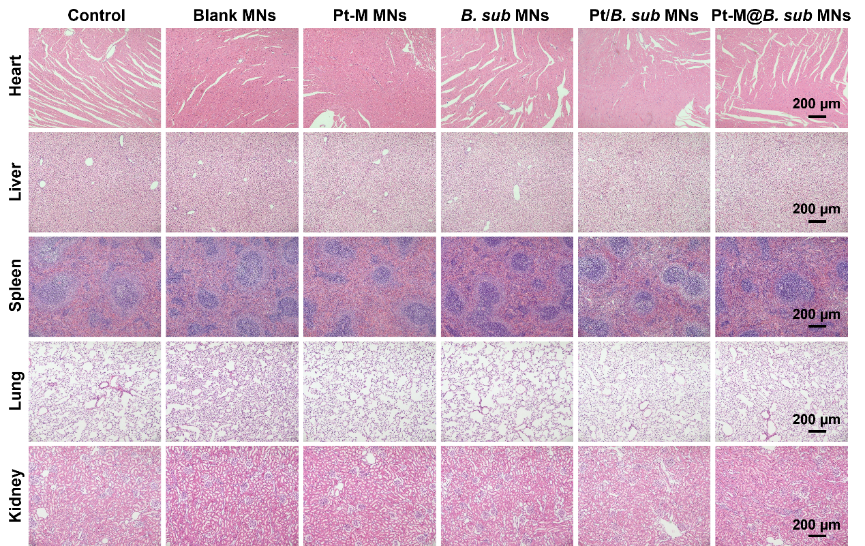


**Figure S17.** Representative H&E staining images of heart, liver, spleen, lung, and kidney harvested from rats on day 15 after different treatments.


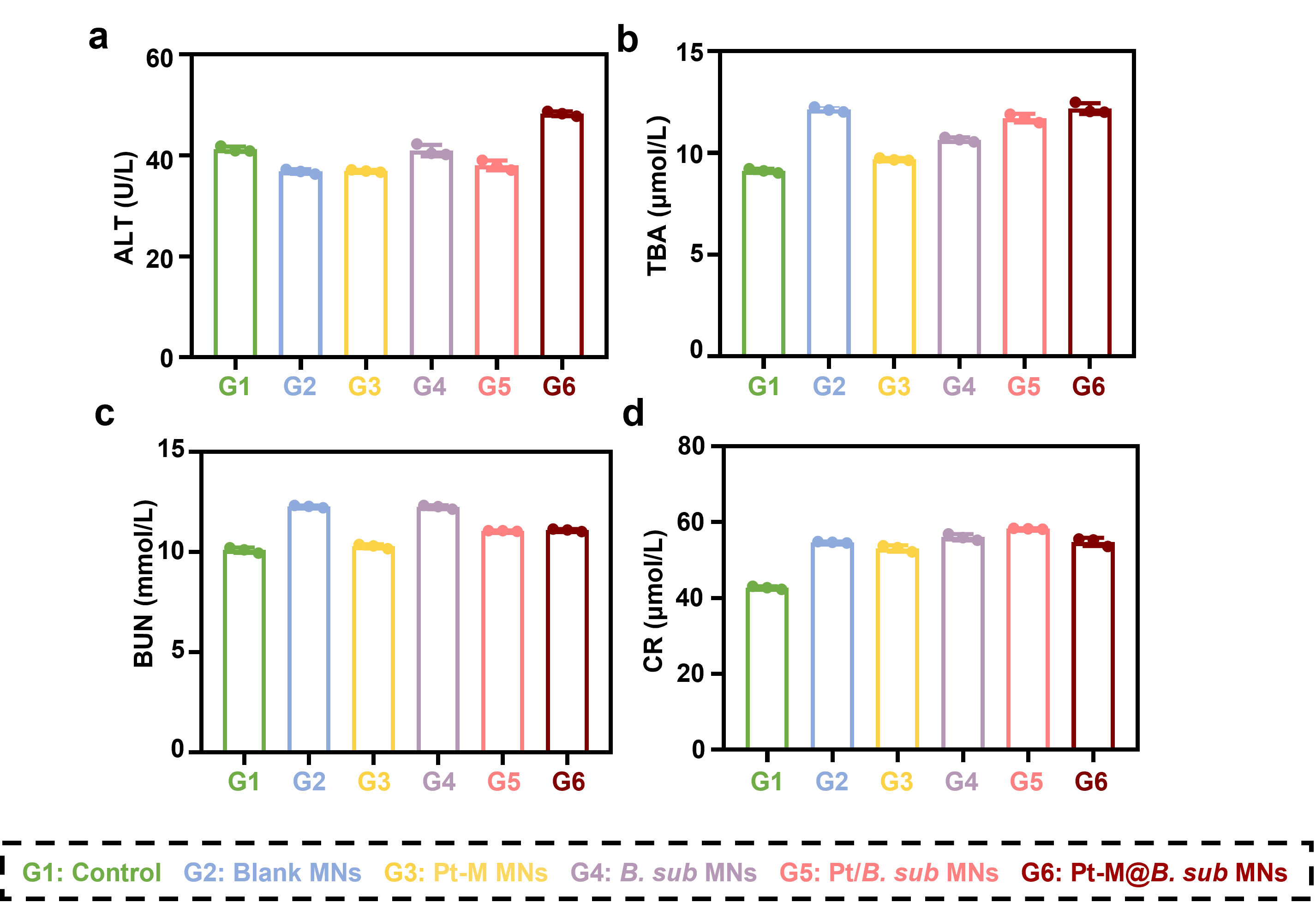


**Figure S18.** Serum biochemical indexes in rats. Serum biochemical indicators indexes of (**a**) alanine transaminase (ALT), (**b**) total bile acid (TBA), (**c**) blood urea nitrogen (BUN) and (**d**) creatinine (CR) of rats after different treatments. Each point represents mean ± SD (n = 3).
